# Dissecting Molecular Evolution in the Highly Diverse Plant Clade Caryophyllales Using Transcriptome Sequencing

**DOI:** 10.1093/molbev/msv081

**Published:** 2015-04-02

**Authors:** Ya Yang, Michael J. Moore, Samuel F. Brockington, Douglas E. Soltis, Gane Ka-Shu Wong, Eric J. Carpenter, Yong Zhang, Li Chen, Zhixiang Yan, Yinlong Xie, Rowan F. Sage, Sarah Covshoff, Julian M. Hibberd, Matthew N. Nelson, Stephen A. Smith

**Affiliations:** ^1^Department of Ecology & Evolutionary Biology, University of Michigan; ^2^Department of Biology, Oberlin College, Science Center K111, Oberlin, OH; ^3^Department of Plant Sciences, University of Cambridge, Cambridge, United Kingdom; ^4^Department of Biology, University of Florida; ^5^Florida Museum of Natural History, University of Florida; ^6^Genetics Institute, University of Florida; ^7^Department of Biological Sciences, University of Alberta, Edmonton, AB, Canada; ^8^Department of Medicine, University of Alberta, Edmonton, AB, Canada; ^9^BGI-Shenzhen, Beishan Industrial Zone, Yantian District, Shenzhen, China; ^10^Department of Ecology and Evolutionary Biology, University of Toronto, Toronto, ON, Canada; ^11^School of Plant Biology, The University of Western Australia, Crawley, WA, Australia

**Keywords:** Caryophyllales, substitution rate heterogeneity, paleopolyploidy, RNA-seq

## Abstract

Many phylogenomic studies based on transcriptomes have been limited to “single-copy” genes due to methodological challenges in homology and orthology inferences. Only a relatively small number of studies have explored analyses beyond reconstructing species relationships. We sampled 69 transcriptomes in the hyperdiverse plant clade Caryophyllales and 27 outgroups from annotated genomes across eudicots. Using a combined similarity- and phylogenetic tree-based approach, we recovered 10,960 homolog groups, where each was represented by at least eight ingroup taxa. By decomposing these homolog trees, and taking gene duplications into account, we obtained 17,273 ortholog groups, where each was represented by at least ten ingroup taxa. We reconstructed the species phylogeny using a 1,122-gene data set with a gene occupancy of 92.1%. From the homolog trees, we found that both synonymous and nonsynonymous substitution rates in herbaceous lineages are up to three times as fast as in their woody relatives. This is the first time such a pattern has been shown across thousands of nuclear genes with dense taxon sampling. We also pinpointed regions of the Caryophyllales tree that were characterized by relatively high frequencies of gene duplication, including three previously unrecognized whole-genome duplications. By further combining information from homolog tree topology and synonymous distance between paralog pairs, phylogenetic locations for 13 putative genome duplication events were identified. Genes that experienced the greatest gene family expansion were concentrated among those involved in signal transduction and oxidoreduction, including a cytochrome P450 gene that encodes a key enzyme in the betalain synthesis pathway. Our approach demonstrates a new approach for functional phylogenomic analysis in nonmodel species that is based on homolog groups in addition to inferred ortholog groups.

## Introduction

Transcriptome sequencing, or RNA-seq, has shown huge potential for understanding the genetic and genomic bases of diversification in nonmodel systems (e.g., Barker et al. 2008; Dunn et al. 2008; Lee et al. 2011; Wickett et al. 2011; Delaux et al. 2014; Li et al. 2014; Misof et al. 2014; Sveinsson et al. 2014; Wickett et al. 2014; Cannon et al. 2015; Hollister et al. 2015). Previous phylogenomic studies based on transcriptomes have been limited by availability of data sets that include both high numbers of genes and dense taxon sampling. Methodological issues in homology and orthology inference, especially in accommodating the frequent genome duplications in plants, have resulted in the discarding of a large proportion of genes from previous phylogenomic studies (Yang and Smith 2014). These limitations, together with the dynamic nature of gene expression, gene duplication and loss, lineage-specific heterogeneity in substitution rates, and gene tree topology discordance have resulted in sparse matrices among nuclear genes in prior analyses, leading researchers to reduce data sets to a small number of genes for analysis. These challenges have limited many RNA-seq phylogenomic studies to inferring a species tree and only a small number of studies have explored transcriptome-wide functional analyses beyond one-to-one orthologs or genes involved in a particular functional category (Barker et al. 2008; Lee et al. 2011).

We use recently developed tree-based homology inference methods (Yang and Smith 2014) that overcome many of the previous analytical limitations to illustrate the rich content of transcriptome data sets. We demonstrate our approach in the plant clade Caryophyllales by applying these methods to a data set of 69 transcriptomes and 27 genomes. With an estimated 11,510 species in 34 families (APG III; Bremer et al. 2009), the Caryophyllales represent approximately 6% of angiosperm species diversity, and are estimated to have a crown age of approximately 67–121 Ma (Bell et al. 2010; Moore et al. 2010). Species of the Caryophyllales are found across all continents and in all terrestrial ecosystems and exhibit extreme life-history diversity, ranging from tropical trees to temperate annual herbs, and from long-lived desert succulents such as cacti to a diverse array of carnivorous plants (e.g., sundews [*Drosera*] and Old World pitcher plants [*Nepenthes*]). The group also contains several independent origins of C_4_ and CAM photosynthesis and exhibits repeated adaptation to warm, dry, and high salinity environments (Sage et al. 2011; Edwards and Ogburn 2012; Kadereit et al. 2012). The extraordinary diversity in growth forms and ecological adaptations makes Caryophyllales an ideal group for investigating gene and genome evolution and heterogeneity in molecular substitution rate.

We apply a tree-based homology and orthology inference approach (Yang and Smith 2014) to examine phylogenetics, molecular substitution rate, and gene and genome duplications in the Caryophyllales. We use the inferred ortholog groups to reconstruct the phylogenetic relationships among major clades of Caryophyllales and to evaluate the heterogeneity in phylogenetic signal among ortholog groups. We then use homolog trees to explore patterns of substitution rate heterogeneity among lineages within the Caryophyllales. Previous studies have linked variations in among-lineage substitution rates to a wide range of factors, such as temperature, UV radiation, water limitation, woody versus herbaceous habit, parasitism, speciation rate, generation time, metabolic rate, and rates of mitosis (Barraclough and Savolainen 2001; Davies et al. 2004; Kay et al. 2006; Wright et al. 2006; Smith and Donoghue 2008; Goldie et al. 2010; Gaut et al. 2011; Buschiazzo et al. 2012; Bromham et al. 2013; Lanfear et al. 2013). However, these studies have been based on either a small number of genes and (typically) the use of introns, or the estimation of absolute rates between a few distantly related species pairs (Yue et al. 2010; Buschiazzo et al. 2012). Studies utilizing nuclear gene sequences have also suffered from sparse taxon sampling, where large phylogenetic distances separate the ingroup from a small number of distant outgroups. In this study, we leverage our high gene content and more thorough taxon sampling to test whether substitution rate between woody and herbaceous lineages is uniform across thousands of genes throughout the Caryophyllales. We also map gene duplication events inferred from homolog trees onto the species tree, revealing a number of previously unknown putative genome duplication events. Finally, we discuss the functional categories of genes that experienced high levels of gene family expansion within the Caryophyllales.

## Results and Discussion

### Tree-Based Homology and Orthology Inferences

Our data set comprises 69 Caryophyllales transcriptomes and 27 eudicot genomes. We took advantage of the availability of fully annotated genomes in eudicots that provide high-quality outgroups for rooting the Caryophyllales (Goodstein et al. 2012). The homology inference first used similarity scores (*E* values from BLASTP) to infer putative homolog groups (Yang and Smith 2014). For each putative homolog group, a multiple sequence alignment and a phylogenetic tree were inferred. Spurious branches were then pruned, and a refined alignment and tree were reestimated. From the refined tree, sequence isoforms that form monophyletic or paraphyletic tips were removed. The remaining trees are the homolog trees (Yang and Smith 2014). A homolog group represents a single gene or gene family, or a monophyletic clade within a larger gene family. A total of 10,960 homolog groups were obtained, each consisting of at least 8 of the 69 Caryophyllales taxa.

**Fig. 1.**
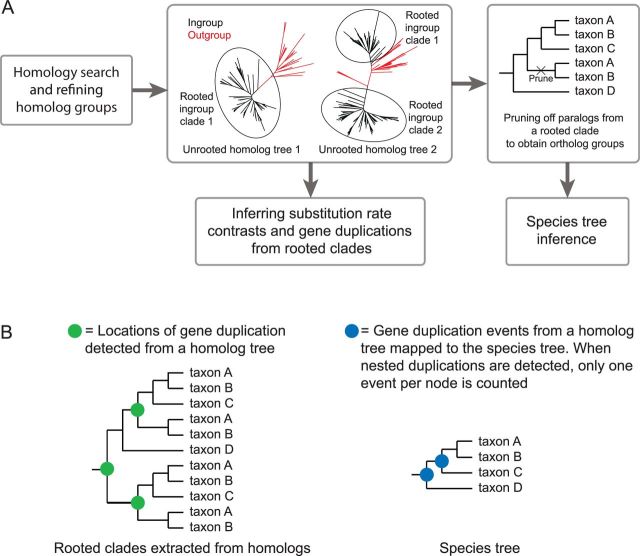
Schematic outline of analyses in this study. (*A*) Homology and orthology inferences and (*B*) mapping gene duplications detected from homolog trees to the species tree.

**Fig. 2.**
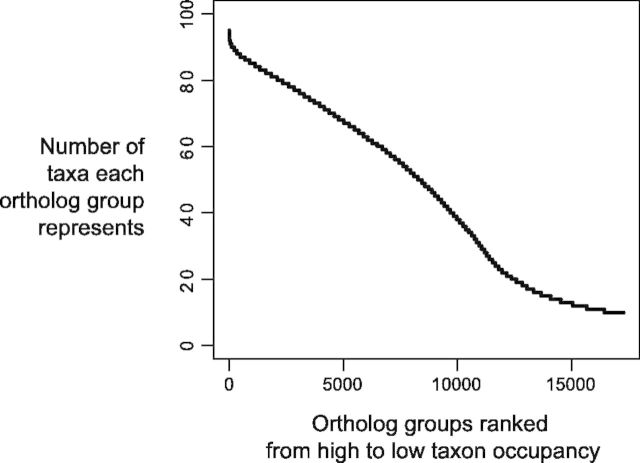
Ortholog groups ranked from high to low by number of taxa represented. Only ortholog groups represented by at least ten Caryophyllales taxa were shown. An ortholog group with full taxon occupancy included 95 taxa.

To estimate a species tree, we further decomposed unrooted homolog trees from amino acid sequences by extracting clades rooted by the earliest diverging species in our data set, *Aquilegia coerulea* (see Materials and Methods), and separating paralogs by inferring the location of gene duplication and pruning the subclade with the smaller number of taxa (fig. 1*A*; Yang and Smith 2014). In this manner, we recovered 17,273 ortholog groups with ten or more Caryophyllales taxa. The taxon occupancy curve (number of taxa per ortholog group ranked from high to low) was nearly straight (fig. 2), indicating that a moderate number of ortholog groups have high taxon coverage. Because *A. coerulea* was used for rooting, it was not present in the ortholog groups. An ortholog group with full taxon coverage therefore consisted of 95 taxa.

### Phylogenetic Relationships in Caryophyllales

We employed two alternative strategies for estimating the species tree and evaluating conflicting phylogenetic signals among genes (i.e., ortholog groups). We first constructed concatenated supermatrices, estimated the maximum-likelihood (ML) phylogeny using RAxML, partitioning by each gene (CA-ML; Stamatakis 2006), and evaluated conflicting phylogenetic signal among genes by jackknife subsampling 10% and 30% of total number of genes (CA-JK10 and CA-JK30; Yang and Smith 2014). We chose the values of 10% and 30% to test the sensitivity of the topology to different ratios of subsampling. In addition to jackknife subsampling, we also carried out fast bootstrap on the supermatrices for comparison (CA-BS). Alternatively, we searched for the Maximum Quartet Support Species Tree (MQSST) using ASTRAL (Mirarab et al. 2014), starting from ML trees estimated from individual ortholog groups. Tree uncertainty was then evaluated using a two-stage bootstrap procedure that first resampled genes and then resampled characters in each resampled gene (MQSST-BS; Seo et al. 2005; Seo 2008). By applying bootstrapping and jackknifing, two nonparametric strategies, we were able to avoid making any assumption on sources of conflict, and we were therefore able to accommodate conflicting phylogenetic signal from potential sources such as hybridization, incomplete lineage sorting, errors, redundancy, and missing data.

We conducted both CA-ML and MQSST analyses on a larger, 1,122-gene data set that included ortholog alignments with at least 85 taxa and 150 columns after trimming, and a smaller, 209-gene data set that included ortholog alignments with at least 90 taxa and 150 columns after trimming. The concatenated 1,122-gene supermatrix had 504,850 amino acid characters, with gene occupancy of 92.1% and character occupancy of 78.7%. The 209-gene supermatrix had an aligned length of 87,082 characters, gene occupancy of 95.6% and character occupancy of 83.6%. Two taxa (*Plumbago auriculata* and *Pereskia aculeata*) had the lowest gene occupancies, occurring in 12.5% and 27.0% of genes in our 1,122-gene supermatrix, respectively. All remaining taxa were present in over 50% of genes, with the vast majority (83 taxa) present in over 90% of genes (supplementary table S1, Supplementary Material online). In the 209-gene supermatrix, 88 out of the total 95 taxa were present in over 90% of genes (supplementary table S1, Supplementary Material online). Our gene and character occupancies are comparable to typical phylogenetic matrices derived from targeted polymerase chain reaction (Cuénoud et al. 2002; Brockington et al. 2009; Soltis et al. 2011). Hence, our data set sampled the genome broadly while minimizing uncertainties introduced by missing data.

**Fig. 3.**
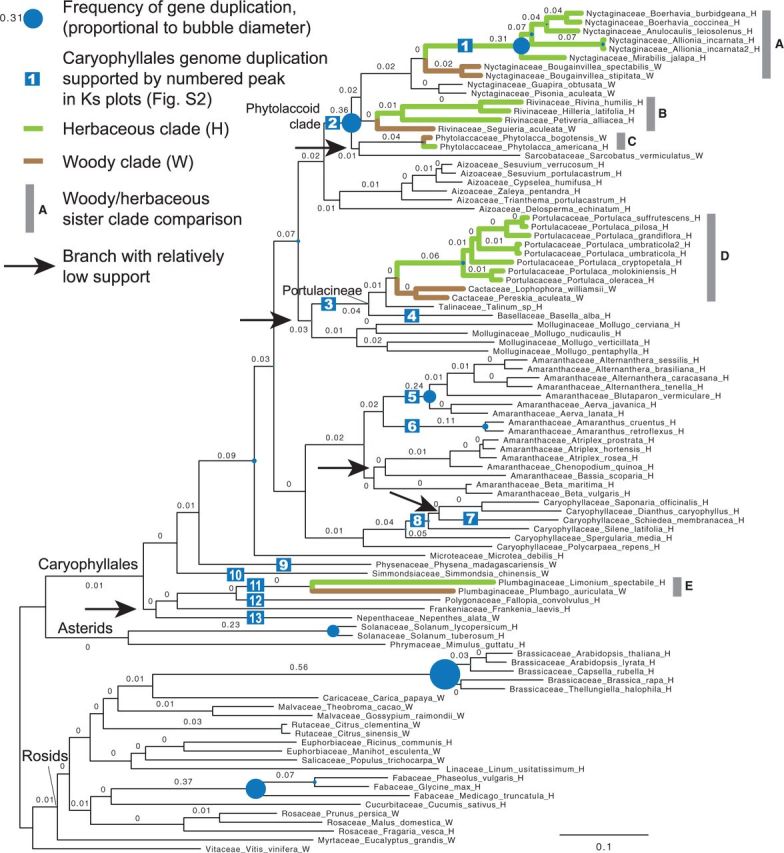
Species tree from RAxML analysis of the 1,122-gene supermatrix. Numbers on branches indicate proportion of genes showing duplication; duplications were not investigated on unmarked branches.

Topologies of both the CA-ML tree and the MQSST based on the 1,122- and 209-gene data sets were well supported overall and were highly congruent with one another and with previous phylogenetic analyses (Cuénoud et al. 2002; Brockington et al. 2009, 2011; Schäferhoff et al. 2009; Soltis et al. 2011; Ruhfel et al. 2014). Most nodes from the 1,122-gene data set and a majority of nodes from the 209-gene data set received 100% support from all analyses (supplementary fig. S1, Supplementary Material online). Only five Caryophyllales branches received CA-JK30 less than 99%, and all five also received MQSST-BS less than 90% (indicated by arrows in fig. 3 and supplementary fig. S1, Supplementary Material online). Below we review nodes that were relatively poorly supported and/or were recovered in incongruent positions.

*Sarcobatus vermiculatus* (Sarcobataceae) of the phytolaccoid clade (which we define here as comprising Nyctaginaceae, Phytolaccaceae s.l. [i.e., including *Agdestis*], Rivinaceae, and Sarcobataceae; Stevens 2015) was recovered in conflicting positions among the four analyses (fig. 3 and supplementary fig. S1, Supplementary Material online). From the 1,122-gene CA-ML analysis, *Sarcobatus* was sister to *Phytolacca* (Phytolaccaceae; 89% BS, 81% JK30, and 59% JK10), whereas the 1,122-gene MQSST (100% BS) and both the 209-gene CA-ML (80%, 61%, and 50%, respectively) and the 209-gene MQSST (89% BS) placed it sister to remaining phytolaccoids. Previous phylogenetic analyses of Caryophyllales have also placed *Sarcobatus* within a polyphyletic Phytolaccaceae with low support (Cuénoud et al. 2002; Brockington et al. 2009, 2011; Schäferhoff et al. 2009). Examination of individual homolog trees revealed that *Sarcobatus vermiculatus* possesses a fast substitution rate compared with its close relatives. Its placement varied among homolog groups and was often poorly supported, with short internodes and low bootstrap percentages. It is possible that additional taxon sampling from Phytolaccaceae would improve support and topological stability for the position of *Sarcobatus*, which represents the only genus (of two closely related species) of Sarcobataceae. In particular, the inclusion of the monotypic genus *Agdestis* (Phytolaccaceae s.l.) would be desirable, as this genus was recovered as sister to *Sarcobatus* with moderate support in previous analyses (Cuénoud et al. 2002; Schäferhoff et al. 2009; Brockington et al. 2011).

Four additional branches within Caryophyllales also had relatively low support (fig. 3 and supplementary fig. S1, Supplementary Material online): 1) The branch uniting Nepenthaceae, Frankeniaceae, Polygonaceae, and Plumbaginaceae, which was topologically congruent with previous analyses (Cuénoud et al. 2002; Brockington et al. 2009, 2011; Soltis et al. 2011) but had moderate to low support values (100% CA-BS/87% CA-JK30/80% CA-JK10/21% MQSST-BS from the 1,122-gene analysis and 95%/77%/54%/32%, respectively, from the 209-gene analysis); 2) the branch uniting Portulacineae with Molluginaceae (100%/95%/77%/87% and 100%/87%/73%/83%, respectively), which was otherwise highly to moderately supported in all previous analyses (Arakaki et al. 2011; Soltis et al. 2011; Brockington et al. 2009, 2011); 3) the branch uniting *Bassia* with the clade of *Chenopodium* + *Atriplex* (Amaranthaceae), which was recovered in each analysis with moderate support (100%/100%/97%/78% and 100%/96%/75%/51%, respectively), whereas previous analyses using chloroplast markers recovered a moderately supported alternative topology of (((*Chenopodium*, *Atriplex*),*Beta*),*Bassia*); and 4) the branch uniting the Caryophyllaceae genera *Schiedea, Silene, Saponaria*, and *Dianthus*, which was weakly supported (88%/67%/56%/66% and 72%/48%/42%/44%, respectively). The former two taxa formed a grade in both the CA-ML and MQSST trees, but switched positions among resampling replicates. Both topologies—(*Schiedea,*(*Silene,*(*Saponaria*, *Dianthus*))) and (*Silene,*(*Schiedea,*(*Saponaria*, *Dianthus*)))—were present in roughly equal numbers of resampling replicates. Previous studies using chloroplast markers, however, recovered a third topology of *Dianthus,*(*Schiedea*, *Silene*) with moderate bootstrap support (Brockington et al. 2011). For both (3) and (4), additional sampling would be needed to distinguish among possible explanations such as ancient or recent hybridization, versus alternative (yet unlikely) explanations such as contamination and mislabeled samples.

Among the non-Caryophyllales eudicots, the placements of *Eucalyptus grandis* and *Cucumis sativus* also had low support values and were in conflict with analyses using chloroplast genomes (Xi et al. 2012; Ruhfel et al. 2014) or genes from both nuclear and organellar genomes (Soltis et al. 2011). In addition, the placement of *Linum usitatissimum* was weakly supported, consistent with previous analyses (Soltis et al. 2011; Xi et al. 2012). Given our focus on Caryophyllales, however, further discussion of these non-Caryophyllales incongruencies lies outside the scope of this article.

A number of processes may account for the relatively low support and/or topological differences observed among the abovementioned nodes, including noise, hybridization, incomplete lineage sorting, and rapid radiation. Concatenation has been criticized for inconsistency when gene trees vary disproportionally in branch lengths (Kolaczkowski and Thornton 2004) and for recovering topologies that are not supported by any gene tree (e.g., Salichos and Rokas 2013). However, coalescence-based methods can also suffer from model violation from both nonrandom missing data and low phylogenetic signal in individual gene trees (Gatesy and Springer 2014; Springer and Gatesy 2014). Complications such as potential hybridization and bias in orthology inference also impact the nature and distribution of the conflicting signal in typical transcriptome data sets and as of yet are unexplored. Therefore, we present the CA-ML and MQSST topologies here because they are derived from the two alternative strategies for species tree estimation that are currently most appropriate for this data set. Also, given the complicated nature of conflict and the processes generating the conflict, species tree uncertainty is best evaluated by nonparametric measurements.

Given the overall high support values, the topological congruencies in the Caryophyllales among results from all four analyses except one single taxon, and the lack of branch length information in MQSST, we use the tree from the 1,122-gene CA-ML as the species tree for subsequent analyses and presentation of results.

### Substitution Rates in Woody versus Herbaceous Lineages

To test the null hypothesis that substitution rate heterogeneity among lineages is not associated with woody versus herbaceous habits, we carried out five woody/herbaceous sister pair comparisons (fig. 3). We extracted rooted Caryophyllales clades that had at least eight taxa from individual homologous gene trees, resulting in a total of 14,165 clades. For each of the five comparisons from each of the extracted Caryophyllales clade, we estimated the synonymous and nonsynonymous branch lengths, assuming that the nonsynonymous (d*N*) to synonymous (d*S*) substitution rate ratio (d*N*/d*S*) was constant among sites and within each woody or herbaceous lineage, but varied freely between the woody and the herbaceous sister lineages. We calculated the substitution rate contrast on each sister pair as the difference in branch lengths between the woody and herbaceous lineage divided by the shorter of the two. We designated the rate contrast to be positive when the substitution rate of the herbaceous lineage was faster than the woody lineage, and negative otherwise (Smith and Donoghue 2008). We used this nonparametric approach to avoid the uncertainties introduced by ancestral state reconstruction and molecular dating, and to avoid complications from gene duplications and conflicting tree topologies in certain areas. In addition, by focusing on nonoverlapping clades, we were able to avoid multiple tests (Smith and Donoghue 2008).

**Fig. 4.**
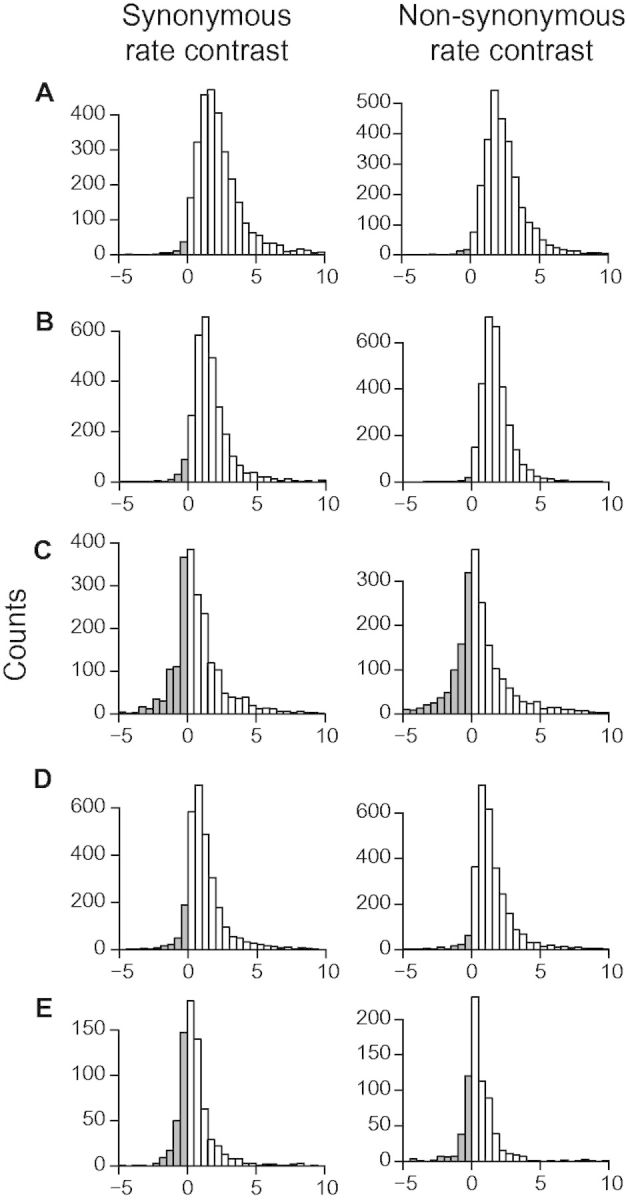
Distribution of substitution rate contrasts. Contrast values were calculated from individual homologous gene trees for five woody–herbaceous sister pairs (*A–E* in fig. 3). Rate contrasts were considered positive (blank) when the herbaceous (H) side possessed a faster rate than the woody (W) wide, and negative (gray) when the reverse was true.

A random subset of 3,000 sister pairs were analyzed for each of the five comparisons (except 722 for contrast E due to the small data set size of *Plumbago auriculata*; fig. 4 and tables 1 and 2). All had more positive than negative contrast values among both synonymous and nonsynonymous sites, and all ten sign tests were highly significant (table 1). To assess a potential node density bias, we tested cases where the woody and herbaceous sister lineage had the same number of tips and found that the pattern holds (table 2; Hugall and Lee 2007). Assuming that the habit of the taxa sister to each contrast was the best approximation of the ancestral habit type, the pattern also holds regardless of whether the direction of the transition was from woody to herbaceous (contrasts A and C, fig. 3), from herbaceous to woody (contrasts D and E), or uncertain (contrast B). The highest rate differences were found in contrast A (herbaceous side with a rate 3.1× as high as the woody side for both d*N* and d*S*). Contrasts C and E both had only one taxon on each side of the comparison and had less pronounced differences in rate compared with contrasts A, B, and D. This is likely related to the fact that contrasts A, B, and D had more taxa than contrasts C and E, which reduced effects of both rate stochasticity and noise introduced by transcriptome sequencing and assembly.

**Table 1.** Substitution Rate Contrasts between Woody (W) and Herbaceous (H) Sister Pairs: Without Filtering by Number of Tips.

	Synonymous Rates				Nonsynonymous Rates			
	No. of W > H	No. of W < H	*P*(W = H)	Median Contrast	No. of W > H	No. of W < H	*P*(W = H)	Median Contrast
Contrast A	49	2,951	<2.2e-16	2.227	68	2,932	<2.2e-16	2.018
Contrast B	31	2,969	<2.2e-16	1.624	150	2,850	<2.2e-16	1.382
Contrast C	1,065	1,917	<2.2e-16	0.564	935	2,008	<2.2e-16	0.605
Contrast D	165	2,835	<2.2e-16	1.176	336	2,664	<2.2e-16	0.904
Contrast E	188	534	<2.2e-16	0.366	231	491	<2.2e-16	0.331

Note.—A random subset of 3,000 extracted Caryophyllales clades were used for each sister pair, except for contrast E, in which we analyzed all clades. Contrast values either lower than −10 or higher than 10 were excluded. *P* values were calculated from sign tests, assuming that the average substitution rates were equal between the woody and the herbaceous side at each sister pair.

**Table 2.** Substitution Rate Contrasts between Woody (W) and Herbaceous (H) Sister Pairs: Values Calculated from Cases Where the Woody Lineage and the Herbaceous Lineage Had the Same Number of Tips.

	Synonymous Rates				Nonsynonymous Rates			
	No. of W > H	No. of W < H	*P*(W = H)	Median Contrast	No. of W > H	No. of W < H	*P*(W = H)	Median Contrast
Contrast A	14	160	<2.2e-16	1.950	22	152	<2.2e-16	1.484
Contrast B	23	874	<2.2e-16	1.434	93	804	<2.2e-16	1.199
Contrast C	1,065	1,917	<2.2e-16	0.564	935	2,008	<2.2e-16	0.605
Contrast D	66	180	2.1e-13	0.715	84	162	7.5e-7	0.461
Contrast E	188	534	<2.2e-16	0.366	231	491	<2.2e-16	0.331

Note.—A random subset of 3,000 extracted Caryophyllales clades were used for each sister pair, except for contrast E, in which we analyzed all clades. Contrast values either lower than −10 or higher than 10 were excluded. *P* values were calculated from sign tests, assuming that the average substitution rates were equal between the woody and the herbaceous side at each sister pair.

We found strong evidence that substitution rates in herbaceous lineages are up to three times as fast as in their woody relatives. The effect is observed in both synonymous and nonsynonymous sites and is most likely driven by differences in mutation rate between woody and herbaceous plants. Our study is the first to confirm such patterns across a large proportion of protein-coding genes in the plant genome using dense taxon sampling. Although the pattern generally holds true across the Caryophyllales, there is one notable exception. The robust shrub *Sarcobatus vermiculatus* exhibits a higher substitution rate and longer branch than any other woody taxon sampled in the phytolaccoid clade, comparable to or exceeding certain herbaceous lineages (fig. 3). The cause for this elevated rate is unclear.

### Ancient Genome Duplications

Of all the homolog trees, 4,420 contained at least one clade with 60 or more Caryophyllales taxa and at least one non-Caryophyllales taxon. From these homolog trees, 167 extracted Caryophyllales clades, 3,651 asterid clades, and 246 rosid clades had average bootstrap support values of at least 80% and were used for mapping gene duplication events. By mapping rooted clades with both high taxon occupancies and high bootstrap support values to the species tree (fig. 1*B*), we detected six branches with highly elevated frequencies of gene duplications (circles in fig. 3 and supplementary fig. S2, Supplementary Material online). The highest concentration of gene duplications occurred at the base of Brassicaceae (fig. 3) where 56% of the genes retained two or more duplicated copies in at least two taxa. The high percentage of duplicate genes reflects the alpha and beta paleotetraploidy events of Brassicaceae (Bowers et al. 2003; de Smet et al. 2013). The second location of elevated gene duplication was found along the branch uniting *Medicago*, *Phaseolus,* and *Glycine* where 37% of genes retained duplicated copies, again corresponding to a known tetraploidy event (Fawcett et al. 2009). As we only recorded gene duplications that shared at least two tips on both sides after trimming tips as a positive control for the Caryophyllales, we had less power for detecting genome duplications involving only two terminal taxa. Still, there was strong signal for genome duplication along the branch leading to *Solanum* in asterids, where 23% of genes retained at least two duplicated copies in both *Solanum lycopersicum* and *Solanum tuberosum*, which corresponds to a known paleohexaploidy event (Tomato Genome Consortium 2012). These three genome duplication events represent all of the previously recognized genome duplications that involved at least two taxa in our data set (Lee et al. 2013).

Using the same approach we detected three previously unrecognized paleopolyploid events in Caryophyllales, corresponding to three branches with highly elevated percentages of genes showing gene duplications (circles in fig. 3 and supplementary fig. S2, Supplementary Material online): At the base of Nyctaginaceae tribe Nyctagineae (represented by *Allionia, Anulocaulis, Boerhavia,* and *Mirabilis*; 31% of genes retaining duplicates), at the base of the phytolaccoid clade (36%), and at the base of the clade within Amaranthaceae containing *Aerva, Alternanthera,* and *Blutaparon* (24%). All three paleopolyploidy events had percentages of gene duplications comparable to the single paleotetraploidy event in Fabaceae. Distribution of synonymous substitutions (Ks plots) also supported the presence and location of all three genome duplication events (peaks 1, 2, and 5, respectively; numbered squares in fig. 3 and supplementary fig. S2, Supplementary Material online). Due to the up to 3-fold synonymous rate heterogeneity between woody and herbaceous clades, peak 2 shifted its location between approximately 0.3 and 1 (supplementary fig. S2, Supplementary Material online). In addition, when peak 1 was present, peak 2 partly overlapped with both peak 1 and the peak between 1.8 and 2 that represents the genome triplication event that occurred in early eudicots (Jiao et al. 2012). Typically, detection of genome duplications has been based on distribution of synonymous distances between paralog pairs, or synteny analyses when genomes are available (Fawcett et al. 2009; Jiao et al. 2011; Jiao et al. 2012; Lee et al. 2013; Sveinsson et al. 2014; Cannon et al. 2015). However, both rate heterogeneity and partially overlapping peaks are known to make detecting genome duplication using the distribution of Ks plots challenging (Vanneste et al. 2013). By incorporating both homolog tree topology and Ks plots, we were able to overcome both challenges and recover previously unrecognized ancient genome duplications.

Six additional peaks (4, 7, 9, 10, 12, and 13) were found, each involving a single terminal taxon (fig. 3 and supplementary fig. S2, Supplementary Material online). These were not detectable using the homolog tree topology as we required at least two terminals to be duplicated to identify a duplicated gene. The lack of a peak at a similar position in the Ks plots of their sister taxa indicates that these seven genome duplication events occurred along each terminal branch. The genome duplication event along the branch leading to *Schiedea* (peak 7) after its split from *Silene latifolia* is further supported by a previous Ks analysis based on expressed sequence tag (EST) (Kapralov et al. 2009). Similarly, both peaks 6 and 11 involve two taxa each and are therefore less detectable from the homolog tree topology alone after masking monophyletic and paraphyletic tips. This is especially true for *Plumbago auriculata*, which is an unusually low-coverage data set (supplementary table S1, Supplementary Material online). The lack of homolog groups showing duplication at the node uniting Plumbaginaceae and Polygonaceae supports that peaks 11 and 12 are from independent genome duplications that happened after this split. In addition, the lack of a Ks peak around 0.8 in *Frankenia* indicates that peak 13 is independent from peaks 11 and 12.

Detecting genome duplications using homolog tree topologies also has less power where ancient and rapid divergences occurred and when individual gene trees had little phylogenetic information. This is likely the case for peaks 3 (Portulacineae) and 8 (within Caryophyllaceae), both near nodes of low support values (fig. 3 and supplementary fig. S2, Supplementary Material online). In both cases, gene duplications found from homolog trees were mapped to the node coincident with the most recent common ancestor of taxa sharing the Ks peak, as well as deeper nodes. Further analyses and additional taxon sampling in Caryophyllaceae are needed to distinguish among possible explanations such as lack of information, incomplete lineage sorting, and/or allopolyploidy.

Earlier phylogenetic analyses have suggested that the backbone of Caryophyllales may have radiated rapidly (e.g., Brockington et al. 2009). Nevertheless, the absence of genome duplications along the deeper branches of Caryophyllales is supported by analyses of the *Beta vulgaris* genome (Dohm et al. 2012, 2014). Comparisons of this genome with the grape genome showed one-to-one synteny and no evidence of lineage-specific genome duplication along the branches leading to *Beta vulgaris* (Dohm et al. 2012, 2014).

We detected 13 genome duplication events within the Caryophyllales. Our methods differ from previous studies that detect genome duplication using transcriptome data (Barker et al. 2008; Sveinsson et al. 2014; Cannon et al. 2015) in that we use homolog tree topology much more extensively to infer the precise phylogenetic locations of peaks in Ks distributions. Although one can apply sophisticated models to overcome saturation and partially overlapping peaks, and to correct for substitution rate heterogeneity among lineages (Vanneste et al. 2013), phylogenetic tree inference incorporates information beyond synonymous distances and therefore is capable of overcoming these challenges effectively.

### Exploring Functional Categories of Genes

Aside from the phylogenetic locations of gene duplications, we also investigated functional categories of genes that showed high taxon occupancy or lineage-specific duplications. We carried out a gene ontology (GO) enrichment analysis for the 4,420 homolog groups that included at least 60 Caryophyllales taxa against a background of all 10,960 homolog groups with at least eight Caryophyllales taxa. The enriched GO terms involved a variety of housekeeping functions such as aminoacyl-tRNA ligase activity, protein targeting to vacuole, Golgi vesicle transport, monosaccharide/oligosaccharide/polysaccharide metabolic process, carbohydrate biosynthetic process, proteolysis, cellular amino acid metabolic process, nucleotide biosynthetic process, response to metal ion, and chloroplast organization (supplementary table S2, Supplementary Material online). The fact that homolog groups with a high number of taxa are enriched for housekeeping functions confirms that these genes are constitutively expressed and are successfully recovered by our homology inference method.

Next, we investigated gene duplicates that can be identified based on phylogenetic trees with the current species sampling (tables 3 and 4). It is important to note two potential issues that likely lead to some level of underestimation of gene duplications in our data. First, our transcriptome data were restricted to certain tissue types at specific developmental stages (mostly leaves), and hence could not capture all gene expression throughout the plant. Second, the fact that de novo assembly often cannot reliably distinguish recent paralogs from allelic variation led to our exclusion of monophyletic and paraphyletic tips that belonged to the same taxon in the homolog trees. This likely also obscured some recent gene duplication events, especially in areas with less taxon sampling. Despite the possible underestimation of the true number of gene duplications, we postulate that if we can detect clear examples of genes that have undergone lineage-specific duplication within the Caryophyllales—that is, if we can recover copies that are coexpressed at levels high enough to detect using de novo assembly—then these duplicated copies may contribute to lineage-specific adaptation.

**Table 3.** Caryophyllales Clades with the Highest Number of Tips for Any Single Taxon.

Clade ID	No. of Tips	No. of Taxa	Taxa with The Most Copies:No. of Copies	Annotation
cc2163-1.mm.cary	105	30	Nyctaginaceae_Anulocaulis_leiosolenus_H:18	Probable nucleoredoxin 1-like (*Citrus sinensis*)
cc123-2.mm.1.cary	354	65	Amaranthaceae_Alternanthera_brasiliana_H:17; Caryophyllaceae_Spergularia_media_H:13	Oxidoreductase family protein (*Populus trichocarpa*)
cc42-2.mm.2.cary	74	23	Nyctaginaceae_Allionia_incarnata2_H:16; Nyctaginaceae_Allionia_incarnata_H:16	Retrotransposon protein, putative, Ty1-copia subclass (*Oryza sativa*, Japonica Group)
cc504-1.mm.1.cary	184	41	Aizoaceae_Sesuvium_ventricosum_H:15; Nyctaginaceae_Allionia_incarnata2_H:13	Mariner transposase (*Beta vulgaris* subsp. *vulgaris*)
cc3-6.mm.1.cary	218	54	Portulacaceae_Portulaca_molokiniensis_H:15	Contains similarity to reverse transcriptases (*Arabidopsis thaliana*)
cc327-1.mm.3.cary	56	18	Amaranthaceae_Atriplex_rosea_H:15	Protein FAR1-related sequence 5-like (*Citrus sinensis*)
cc1593-1.mm.1.cary	56	18	Nyctaginaceae_Boerhavia_burbidgeana_H:14	Hypothetical protein VITISV_026753 (*Vitis vinifera*)
cc3-8.mm.cary	56	18	Nyctaginaceae_Boerhavia_burbidgeana_H:14	Integrase core domain containing protein (*Solanum demissum*)
cc28-1.mm.1.cary	353	68	Nyctaginaceae_Boerhavia_coccinea_H:13	Cytochrome P450 71A25-like (*Fragaria vesca* subsp. *vesca*)
cc6-7.mm.1.cary	99	30	Nyctaginaceae_Boerhavia_coccinea_H:13	Putative reverse transcriptase (*Arabidopsis* t*haliana*)
cc27-3.mm.1.cary	279	62	Amaranthaceae_Aerva_lanata_H:13	G-type lectin S-receptor-like serine/threonine-protein kinase At1g11330-like (*Vitis vinifera*); recognition of pollen
cc1593-1.mm.1.cary	97	18	Nyctaginaceae_Allionia_incarnata2_H:13	Uncharacterized protein LOC100259102 (*Vitis vinifera*)
cc5-17.mm.1.cary	352	59	Nyctaginaceae_Guapira_obtusata_W:13; Amaranthaceae_Atriplex_hortensis_H:13	NBS-LRR type resistance protein (*Beta vulgaris*)

**Table 4.** Caryophyllales Clades with the Highest Total Number of Tips.

Clade ID	No. of Tips	No. of Taxa	Annotation
cc25-1.mm.1.cary	361	68	Cytochrome P450, family 72, subfamily A, polypeptide 15 isoform 2 (*Theobroma cacao*)
cc123-2.mm.1.cary	354	65	Oxidoreductase family protein (*Populus trichocarpa*)
cc28-1.mm.1.cary	353	68	Cytochrome P450 71A25-like (*Fragaria vesca* subsp. *vesc*a)
cc5-17.mm.1.cary	352	59	NBS-LRR type resistance protein (*Beta vulgaris*)
cc333-1.mm.1.cary	302	63	Cytochrome P450 76C1-like (*Vitis vinifera*)
cc144-1.mm.1.cary	287	68	Peptide transporter PTR2-like (*Solanum tuberosum*)
cc171-1.mm.1.cary	285	61	Zeatin O-glucosyltransferase-like (*Fragaria vesca* subsp. *vesca*)
cc27-3.mm.1.cary	279	62	G-type lectin S-receptor-like serine/threonine-protein kinase At1g11330-like (*Vitis vinifera*)
cc521-1.mm.cary	277	67	GDSL esterase/lipase 1-like (*Vitis vinifera*)
cc50-2.mm.1.cary	273	63	Wall-associated receptor kinase-like 9-like (*Vitis vinifera*)

We ranked extracted Caryophyllales clades with at least five taxa by either the highest number of tips for any single taxon (table 3) or the total number of tips (table 4) that can be identified based on our taxon sampling and homolog trees. Of the 13 Caryophyllales clades with the highest copy number for any taxon, five represented transposable elements (table 3). The remaining clades were involved in four functional classes: Oxidoreductase (nucleoredoxin 1-like, cytochrome P450 and other oxidoreductase family proteins), pollen recognition (G-type lectin S-receptor-like serine/threonine-protein kinase), disease resistance signaling (NBS-LRR type resistance protein), and transcription factors (protein FAR1-related sequence 5-like). Most of the taxa with the highest number of tips in homolog trees belong to Nyctaginaceae, likely due to both the denser taxon sampling in Nyctaginaceae and the multiple genome duplication events in the phytolaccoid clade (fig. 3).

To minimize the effect of taxon sampling, we ranked Caryophyllales clades by the total number of tips. Of the ten Caryophyllales clades with the highest total number of tips, three belonged to the cytochrome P450 family (table 4), with the largest clade consisting of 361 tips. Functional categories for these Caryophyllales clades include oxidoreductase, disease resistance, peptide transportation, kinases, and lipid degradation (table 4). Notably, the Caryophyllales clade that was annotated as “Cytochrome P450 71A25-like” encodes a key enzyme in the betalain synthesis pathway (supplementary fig. S3, Supplementary Material online; Hatlestad et al. 2012). Betalains are a group of pigments unique to core Caryophyllales that produce bright yellow, pink and violet colorations in vegetative, flower and fruit tissues, and that have been shown to have strong antioxidant activity (Gandía-Herrero and García-Carmona 2013). Further exploration of the locations of gene family expansion may be useful in identifying recruitment of key candidate genes for future functional dissection of other ecophysiological traits and developmental processes that are frequently encountered in Caryophyllales and are of general interest, such as photosynthetic pathways (Christin et al. 2014), salt tolerance, succulence, and carnivory. Additional, carefully targeted taxon sampling will increase the power of such an approach.

## Conclusion

This study demonstrates a new approach for functional phylogenomic analysis in nonmodel species using homolog groups in addition to inferred ortholog groups. We show that transcriptome data sets can provide high-quality homolog and ortholog sets that are rich resources not only for reconstructing phylogenies but also for mapping substitution rate shifts and gene duplications. The power and utility of these data sets are highly contingent on efficient and accurate clustering, aligning, and cleaning in order to obtain high-quality homolog trees. From these homolog trees, we were able to recover candidate genes that experienced extensive lineage-specific gene family expansion, which provide a rich resource for investigating genes that may have contributed to adaptive changes.

## Materials and Methods

### Data Availability

Raw reads for the six newly generated transcriptomes were deposited in the NCBI Sequence Read Archive (BioProject: PRJNA261527; see supplementary table S3, Supplementary Material online, for individual accessions). Assembled sequences, alignments, and trees are available from Dryad (doi:10.5061/dryad.33m48). Scripts used for analyses in this study are also available from Dryad with notes and updates available from https://bitbucket.org/yangya/caryophyllales_mbe_2015.

### Sampling and Laboratory Procedures

Assembled transcripts and translated amino acid sequences from 66 Caryophyllales transcriptome data sets were obtained from the One Thousand Plants (1KP) Consortium (https://sites.google.com/a/ualberta.ca/onekp/, last accessed April 10, 2015). Each transcriptome was derived from leaves and/or flower buds. RNA isolation, quality control, library preparation, and sequencing procedures were summarized in Johnson et al. (2012), and collection information is available on the 1KP website. After preliminary phylogenomic analysis and examination of gene trees and the matrix occupancy, four 1KP transcriptomes were removed due to contamination or small data set size (supplementary table S1, Supplementary Material online). Of the 62 1KP transcriptomes used in this study, *Bassia scoparia* was published by the 1KP pilot study labeled as its synonym *Kochia scoparia* (Matasci et al. 2014; Wickett et al. 2014). The remaining 61 transcriptomes from 1KP are published here for the first time (supplementary table S1, Supplementary Material online). In addition, the sugar beet (*Beta vulgaris*) EST data were downloaded from The *Beta vulgaris* Resource website (http://bvseq.molgen.mpg.de, last accessed June 23, 2013) (Herwig et al. 2002; Dohm et al. 2012).

We generated six additional transcriptomes for this study (supplementary table S3, Supplementary Material online). Young leaves and/or flower buds were frozen in liquid nitrogen and stored at −80 °C. Total RNA was extracted using the BIO-RAD Aurum Total RNA Mini Kit (Life Science Research, Hercules, CA) and quantified using the NanoDrop 1000 Spectrophotometer (Thermo Scientific, Wilmington, DE). Libraries were prepared using the TruSeq RNA Sample Preparation Kit v2 (Illumina, Inc., San Diego, CA) with modifications (supplementary methods, Supplementary Material online), and quantified on an Agilent 2100 Bioanalyzer (Agilent Technologies, Inc., Santa Clara, CA). All six libraries were multiplexed and sequenced on one lane of an Illumina HiSeq2000 sequencer at the University of Michigan DNA Sequencing Core.

For outgroup comparison, nonredundant transcripts and amino acid sequences for 27 eudicot species derived from genome annotations were downloaded from Phytozome v9 (Goodstein et al. 2012).

### Sequence Processing

Paired end 101-bp reads from the six newly generated transcriptomes were trimmed and filtered using the following procedures: Read pairs with either of the reads having average quality score ≤32 were removed; 3′-end nucleotides with quality scores less than 10 were trimmed; posttrimming read pairs with either of the reads ≤30 bases were removed; and read pairs with adaptor contamination were removed. The remaining reads were assembled using Trinity v2012-02-25 with default settings (Grabherr et al. 2011). Assembled reads were translated using TransDecoder v2012-08-15 with default settings (Haas et al. 2013). Translation of the beet EST sequences was carried out using FrameDP v1.2.0 (Gouzy et al. 2009) with default parameters, except for setting framed_maximum_peptide_length to 1,500, and using a custom blast database consisting of amino acid sequences from the 27 eudicot genome annotations. All translated amino acid sequences from each of the 69 Caryophyllales transcriptome data sets were reduced with CD-HIT v4.6 (-c 0.99 -n 5) (Fu et al. 2012).

### Homology Inference

Initial homology searches were carried out using all-by-all BLASTP with an *E* value cutoff of 10^−^^5^ and -max_target_seqs 100. BLASTP hits with identical matches greater than 50% and query coverage/query length greater than 0.7 were used for homology inference. Putative homolog groups were obtained using Markov clustering (MCL v12-068; van Dongen 2000) with an *E* value cutoff of 10^−^^30^ and an inflation value of 1.4. Only sequences 40 amino acids or longer in length were retained, and only clusters with at least eight Caryophyllales taxa were retained.

Each cluster was aligned using MAFFT v7 (Katoh and Standley 2013) with “–genafpair –maxiterate 1000” (“–auto” for clusters with more than 1,000 sequences), and alignments were trimmed using Phyutility v2.2.6 (Smith and Dunn 2008) with “-clean 0.05.” Phylogenetic trees were estimated using FastTree v2.1.7 (Price et al. 2009, 2010). Because trees with branches longer than two substitutions per site were most likely caused by either error or clusters being pulled together by conserved domains, such long branches were cut, the sequences on each subtree were realigned, the resulting matrix was trimmed, and the phylogeny was reestimated following the same procedure as the initial round until no branch was longer than 2. This process was repeated with a more stringent branch length cutoff of 0.5, set by the distribution of branch lengths among the ingroups. Final alignment was estimated using SATé v2.2.7 with “iter-limit = 3” (Liu et al. 2009, 2012). ML phylogenetic inference was carried out using RAxML v7.3.2 (Stamatakis 2006) with the model PROTCATWAG. Branches greater than 0.6 were cut; spurious terminal branches greater than 0.2 and more than ten times longer than their sisters were also removed (Yang and Smith 2014). Finally, as the transcriptome assembly process produced multiple isoforms for each gene that formed monophyletic or paraphyletic groups, only the one with the highest number of characters in trimmed alignments was retained as the representative, with the rest removed (Yang and Smith 2014). The remaining trees were the homolog trees.

### Orthology Inference and Species Tree Estimation

Clades rooted by the basal eudicot *Aquilegia coerulea* (Ranunculaceae; this species was sister to all others in our analyses) were extracted from homolog trees. For each extracted clade, gene duplication events were inferred by the presence of one or more duplicated taxa between two sides; the side with the smaller number of taxa was subsequently pruned (fig. 1*A*). This procedure was carried out from root to tips iteratively on all subclades until no taxon duplication was present. The alignment for each ortholog group was constructed by extracting aligned sequences from the homologs and trimming by Phyutility with “-clean 0.3” (Smith and Dunn 2008). After visualizing taxon occupancy statistics of ortholog groups, a supermatrix was constructed by concatenating trimmed ortholog alignments with at least 90 taxa and 150 amino acids. A second supermatrix was constructed using ortholog groups with at least 85 taxa and at least 150 amino acids after trimming. A ML tree was estimated from each supermatrix using RAxML with the PROTCATWAG model, partitioning each ortholog group. Node support was evaluated by 200 fast bootstrap replicates. To explicitly evaluate the conflicting phylogenetic signal among ortholog groups, we also carried out 200 jackknife replicates for both supermatrices, randomly subsampling 30% and 10% of the total numbers of genes without replacement, while keeping each gene region intact (Yang and Smith 2014).

In addition to the concatenated analyses, we also searched for the MQSST using ASTRAL v4.7.3 (Mirarab et al. 2014). An ML tree for each ortholog group that had at least 85 taxa and 150 characters after trimming was estimated using RAxML with the PROTCATWAG model. Phylogenetic uncertainty within each ortholog group was estimated using 200 fast bootstrap replicates in RAxML. Uncertainty for the MQSST was estimated with 100 bootstrap replicates using a two-stage multilocus bootstrap strategy (Seo et al. 2005; Seo 2008) as implemented in ASTRAL. A second ASTRAL analysis was carried out with the same setting using ortholog groups that had at least 90 taxa and 150 characters after trimming.

### Substitution Rate Contrasts

To investigate the relationship between habit (woody vs. herbaceous) and substitution rates, we first plotted the habit of all sampled Caryophyllales taxa to the species tree (fig. 3). Although the cactus *Lophophora williamsii* is not a typical tree or shrub, it is similar to typical woody plants in having prominent and persistent above ground tissue and was therefore treated as woody.

We extracted rooted Caryophyllales clades with at least eight taxa from individual homologous gene trees. For each of these Caryophyllales clades, all corresponding amino acid sequences were pooled, and an alignment was estimated using PRANK v140110 (Löytynoja and Goldman 2010) with the default settings except that a single iteration was carried out using the extracted clade as the guide tree. Each resulting alignment was back translated using PAL2NAL v14 (Suyama et al. 2006), and the codon alignment was trimmed by requiring each codon column to have a minimum of 20% unambiguous codons. Both the extracted Caryophyllales clades and the trimmed codon alignments were used to estimate the synonymous (d*S*) and nonsynonymous (d*N*) substitution rates in CODEML, as part of the PAML package v4.8a (Yang 2007). For each of the five woody/herbaceous pairs in a given Caryophyllales clade, we labeled the woody clade as clade #1, its sister herbaceous clade as clade #2, and the rest of the tree as the “background.” Ratios of d*N*/d*S* for the background, clade #1 and clade #2 were estimated separately with constant d*N*/d*S* across sites (model = 2, NSites = 0) and codon frequencies estimated by F3x4. The synonymous branch length contrasts at a given internal node were calculated by stepwise averaging of the corresponding synonymous branch lengths estimated by CODEML from the tips to the node, and dividing the difference in branch lengths between the two sister clades by the shorter of the two (Smith and Donoghue 2008). Similarly, the nonsynonymous contrast was calculated using the corresponding nonsynonymous branch lengths estimated by CODEML. For all woody/herbaceous sister pairs, if the herbaceous side had longer average branch lengths, we designated the contrast values to be positive; values were negative if the woody side had longer branch lengths.

### Inferring the Phylogenetic Location of Gene Duplications

Homolog trees that contained clades with at least one outgroup and 60 ingroup taxa were subject to 100 rapid bootstrap replicates in RAxML to evaluate branch support. Caryophyllales clades containing at least 60 of the 69 taxa, asterid clades containing all three taxa and rosid clades containing at least 18 of the 22 taxa were extracted from homolog trees. Extracted clades with average bootstrap support values ≥80% were used for locating gene duplication events. A gene duplication event was recorded at a node on the extracted clade if the two branches from this node shared two or more taxa. Duplication events from each extracted clade were mapped to the corresponding node on the species tree. When the gene tree had missing taxa or had a conflicting topology compared with the species tree, we mapped the duplication event onto the corresponding most recent common ancestor on the species tree. When nested duplication events were detected from an extracted clade, only one duplication event was mapped to each corresponding node on the species tree for each extracted clade (fig. 1*B*).

### Distribution of Synonymous Distances (Ks) between Paralogous Gene Pairs

To verify putative genome duplications detected from homolog tree topology, we visualized the distribution of Ks within each of the 69 Caryophyllales taxa. The recently published peptides and coding sequences (CDS) from the *Beta vulgaris* genome annotation (Dohm et al. 2014) were used instead of the EST sequence for the Ks analysis. The 1KP accession HURS was used instead of KJAA (combined analysis of accession HURS and BZMI) for the Ks analysis due to the discovery that BZMI is a mixture of *Mollugo pentaphylla* and *M. verticillata*. For all 69 Caryophyllales transcriptome data sets, highly similar sequences were reduced with CD-HIT (-c 0.99 -n 5). An all-by-all BLASTP was carried out within each taxon using an *E* value cutoff of 10 and -max_target_seq set to 20. Resulting hits with pident less than 20% or niden less than 50 amino acids were removed. Sequences with ten or more hits were removed to avoid overrepresentation of gene families that experienced multiple recent duplications. The remaining paralog pairs and their corresponding CDS were used to calculate Ks values using the pipeline https://github.com/tanghaibao/bio-pipeline/tree/master/synonymous_calculation (accessed November 29, 2014). The pipeline first carries out pairwise protein alignment using default parameters in ClustalW (Larkin et al. 2007), back-translates the alignment to a codon alignment using PAL2NAL, and calculates the synonymous substitution rate (Ks) using yn00 as part of the PAML package, with Nei–Gojobori correction for multiple substitutions (Nei and Gojobori 1986).

### Functional Annotation

We obtained the *Arabidopsis thaliana* gene that was most closely related to the Caryophyllales clade of interests based on homolog tree topology and used its locus ID to represent the Caryophyllales clade in the GO analysis. When multiple *A. thaliana* genes are present in the clade sister to the Caryophyllales, a random one was chosen. GO enrichment analysis was carried out using GOrilla (Eden et al. 2009) with control for False Discovery Rate (Benjamini and Hochberg 1995).

In addition to automated annotation for GO enrichment analysis, we also manually annotated clades having the most gene family expansion. Peptide alignments were visually examined, and a sequence with high completeness in the alignment was BLASTed against the nonredundant protein database in NCBI. Functional annotations of top BLAST hits were performed using the UniProt database (http://www.uniprot.org/, last accessed April 18, 2014).

## Supplementary Material

Supplementary methods, figures S1–S3, and tables S1–S3 are available at *Molecular Biology and Evolution* online (http://www.mbe.oxfordjournals.org/).

Supplementary Data
