# Supplementary material for: Dissecting Molecular Evolution in the Highly Diverse Plant Clade Caryophyllales Using Transcriptome Sequencing
Source: Mol Biol Evol. 2015 Apr 2;32(8):2001–14. doi: 10.1093/molbev/msv081 (PMC4833068; doi:10.1093/molbev/msv081)
Supplement: Supplementary Data [file supp_32_8_2001__index.html]

Dissecting Molecular Evolution in the Highly Diverse Plant Clade Caryophyllales Using Transcriptome Sequencing — Dissecting Molecular Evolution in the Highly Diverse Plant Clade Caryophyllales Using Transcriptome Sequencing — Supplementary Data 

# Dissecting Molecular Evolution in the Highly Diverse Plant Clade Caryophyllales Using Transcriptome Sequencing

## Supplementary Data

files

**Files in this Data Supplement:**

- Supplementary Data - pdf file
