# Supplementary material for: Dissecting Molecular Evolution in the Highly Diverse Plant Clade Caryophyllales Using Transcriptome Sequencing
Source: Mol Biol Evol. 2015 Apr 2;32(8):2001–14. doi: 10.1093/molbev/msv081 (PMC4833068; doi:10.1093/molbev/msv081)

Supplementary Methods:
Notes and modifications to the Illumina TruSeq v2 protocol

Courtesy of Richard Cronn, US Forest Service Pacific Northwest Research Station
with notes from Ya Yang and Michael J. Moore, May 2012

* Indicates added directional RNA-seq prep steps

Reagents/equipment:

Item	Catalog Number	Size
Illumina TruSeq RNA Sample preparation kit	RS-122-2001	48 rxn
Superscript III first strand synthesis kit	18080-051	50 rxn
NEBNext mRNA Second Strand Synthesis enzyme mix	NEB E6111S	20 rxn
NEBNext Second Strand Synthesis Buffer, dNTP-Free	NEB B6117S	0.4 ml
USER (uracil-specific excision reagent) Enzyme mix	NEB M5505S	50 Units
2 mM dNTP/ 4 mM dUTP Mix	FERR0251	1 ml
Sephadex G-50	G50150-50G	10 g
Millipore Multiscreen Column Loader (EMD Millipore)	MACL09645	1
Millipore MultiScreen 96-Well Assay Plates for Sample Preparation	MAHVN4510	Pack of 10

Preparations:

1. *Hydrate Sephadex.
   a. Add dry Sephadex G-50 to Millipore column loader. Remove excess resin from the top of the column loader with the supplied scraper.
   b. Place the multiscreen HV plate upside-down on top of the column loader and invert both the multiscreen HV plate and the column loader.
   c. Tap on the top of the column loader to release the resin.
   d. Add 300 µL of ddH2O to each well. Let stand at room temperature for 3 hours to allow resin to swell. Once resin has swollen in the multiscreen plates, these plates can be sealed with Saran Wrap and stored at 4°C for several weeks in a sealed plastic container containing a damp paper towel to assure they are kept moist.

2. Quantify RNA and use approximately 3 µg total RNA as starting material.

3. Before starting, spray and clean bench top, pipettes, and the centrifuge with RNase Zap.

Modifications to the TruSeq v2 protocol:

1. Page 45 step 5–6 of "Make CDP": add 7 µL of First Strand Master Mix and 1 uL of SuperScript II to each tube.

*After finishing "incubate 1 CDP" on page 45, instead of proceeding to the next step, do the following:
   a. Place the G50-containing Multiscreen filter plate on top of a 96-well receiver. Stabilize this "sandwich" with 2 pieces of lab tape.
   b. Centrifuge the sandwich for 5 minutes at 2500 rpm (1000 x g) at room temperature. This packs the columns and removes excess water.

c. Undo sandwich and remove the water in the 96-well receiver. Re-make sandwich and add 150 L dH2O to each well in the G50-containing Multiscreen filter plate and spin for an additional 5 min at 2500 rpm at room temperature.
d. Disassemble the sandwich and discard the water that collected in the 96-well receiver.
e. Add samples to the G50-containing Multiscreen filter plate, making sure to not disturb the resin (minimum volume is 10 μL; max volume is 100 μL).
f. Place the G50-containing Multiscreen filter plate on top of a new 96-well receiver plate. Stabilize this "sandwich" with 2 pieces of lab tape.
g. Spin at 2500 rpm for 5 min at room temperature; spin again if filtered volume is less then 20 μL.
h. Transfer the 20 μL samples to new PCR tubes.
i. Per sample, mix the following reagents from the Superscript III kit together in a master mix:

- 1 ul 10X Reverse Transcription buffer
- 2 ul 25 mM MgCl2
- 1 ul 0.1 M DTT
- 0.5 μL random hexamers (50 ng/μL)

Add 4.5 μL of the master mix to each well and mix by pipetting gently.
Per sample, mix the following into a master mix:

- 4 μL of 10X dNTP-FREE Second Strand buffer (NEB B6117S)
- 5 μL dNTP/dUTP mixture
- 2 μL Second Strand enzyme mix (NEB E6111S)

Pipette 11 μL of the master mix into each resuspended RNA to give a final volume 40 μL.
Incubate 2 hours at 16°C.
Continue protocol as directed (page 47, Purify CDP). Note that the volume of sample is different from following the TruSeq protocol. Add 64 μL AMPure beads to each sample (sample volume = 35.5 μL; keep sample:beads = 1:1.8 to get all DNA)

2. *Prior to PCR enrichment, degrade the dUTP in the coding strand (new step)
a. Per sample, add 1 μL of USER enzyme mixture.
b. Incubate at 37°C for 15 minutes, then place on ice. Continue to PCR enrichment step

3. During the PCR enrichment step, set up the PCR program so that temperature change is no faster than 2°C per min.

4. After PCR enrichment, check PCR products on a 1.5% agarose gel before running the library on a 2100 Bioanalyzer (Agilent Technologies, Inc.).

Comments added by Ya Yang Jan 2014: This stranded protocol adds 3–4 hours to the original TruSeq v2 protocol. The resulting strand specificity is lower than that observed when using the Illumina TruSeq Stranded mRNA Sample Prep Kit, which was released subsequent to the lab work described in this manuscript. We have since switched to the latter kit for the lower total cost, shorter prep time and better strand specificity. Due to the low strand specificity, we used the default, non-stranded method for *de novo* assembly using Trinity for the six newly generated transcriptomes.

Table S1. Taxa included in this study and their occupancies in the supermatrices, ranked from high to low gene occupancy in the 1,122-gene matrix. Taxon codes are the shortened taxon names used throughout the analyses. All 1KP data are published here for the first time except *Bassia scoparia*, which was published under its older synonym *Kochia scoparia* in Matasci *et al.* (2014) and Wickett *et al.* (2014) with SRA accession ERR364385. Two Caryophyllales taxa from 1KP were removed from the analyses due to their small data set sizes: *Ancistrocladus tectorius* (HSXO) and *Opuntia* sp. (QAIR). Two additional data sets from 1KP were removed due to contamination: *Tamarix chinensis* (HTDC) and *Amaranthus palmeri* (JBGU). A fifth 1KP data set labeled as *Mollugo pentaphylla* (KJAA) was discovered as a mixture of *M. pentaphylla* and *M. verticillata* after we finished the phylogenetic analysis. Since this mixture only affects topology within *Mollugo* we chose not to repeat the entire analysis. One additional taxon from Phytozome [*Aquilegia coerulea* (Acoe)] was used for rooting the remaining taxa and was not included in the final ortholog sets.

**Taxon**	**Source**	**1KP accession**	**Abbreviated Taxon ID**	**1122-gene matrix**				**209-gene matrix**			
				# orthologs	# characters	% orthologs	% characters	# orthologs	# characters	# orthologs	% characters
Arabidopsis thaliana	Phytozome		Atha	1115	481788	99.38%	95.43%	208	83149	99.52%	95.48%
Gossypium raimondii	Phytozome		Grai	1113	487078	99.20%	96.48%	209	84919	100.00%	97.52%
Manihot esculenta	Phytozome		Mesc	1113	466385	99.20%	92.38%	209	80119	100.00%	92.00%
Phaseolus vulgaris	Phytozome		Pvul	1113	480057	99.20%	95.09%	209	83463	100.00%	95.84%
Theobroma cacao	Phytozome		Tcac	1113	487658	99.20%	96.59%	209	84878	100.00%	97.47%
Populus trichocarpa	Phytozome		Ptri	1111	485687	99.02%	96.20%	209	84719	100.00%	97.29%
Citrus clementina	Phytozome		Ccle	1109	479644	98.84%	95.01%	209	83989	100.00%	96.45%
Glycine max	Phytozome		Gmax	1109	483131	98.84%	95.70%	209	84078	100.00%	96.55%
Amaranthus cruentus	1KP	XSSD	XSSD	1106	455446	98.57%	90.21%	207	80599	99.04%	92.56%
Atriplex prostrata	1KP	AAXJ (EPVF+ MUCT)	AAXJ	1105	451580	98.48%	89.45%	208	80192	99.52%	92.09%
Prunus persica	Phytozome		Pper	1105	475929	98.48%	94.27%	209	83908	100.00%	96.36%
Thellungiella halophila	Phytozome		Thal	1103	476224	98.31%	94.33%	208	83076	99.52%	95.40%
Arabidopsis lyrata	Phytozome		Alyr	1102	475403	98.22%	94.17%	207	82706	99.04%	94.97%
Sesuvium verrucosum	1KP	EDIT (OPZX+ ZJDK)	EDIT	1102	461520	98.22%	91.42%	209	81619	100.00%	93.73%
Atriplex hortensis	1KP	ONLQ (BDIW+ AKTA)	ONLQ	1101	458059	98.13%	90.73%	209	80757	100.00%	92.74%
Brassica rapa	Phytozome		Brap	1100	467184	98.04%	92.54%	207	81472	99.04%	93.56%
Atriplex rosea	1KP	CBJR (LBZM+ PDXY)	CBJR	1100	452119	98.04%	89.56%	207	80183	99.04%	92.08%
Guapira obtusata	This study		ILU3	1100	459871	98.04%	91.09%	208	81508	99.52%	93.60%
Solanum lycopersicum	Phytozome		Slyc	1098	467197	97.86%	92.54%	208	82329	99.52%	94.54%
Capsella rubella	Phytozome		Crub	1097	471433	97.77%	93.38%	208	82710	99.52%	94.98%
Bougainvillea stipitata	This study		ILU6	1096	447315	97.68%	88.60%	208	79089	99.52%	90.82%
Linum usitatissimum	Phytozome		Lusi	1096	473369	97.68%	93.76%	207	83011	99.04%	95.33%
Ricinus communis	Phytozome		Rcom	1096	464230	97.68%	91.95%	209	82024	100.00%	94.19%
Eucalyptus grandis	Phytozome		Egra	1094	475502	97.50%	94.19%	206	82985	98.56%	95.30%
Mimulus guttatus	Phytozome		Mgut	1093	462240	97.42%	91.56%	207	81584	99.04%	93.69%
*Allionia incarnata*2	1KP	HMFE (EGOS+ DVXD)	HMFE	1091	428748	97.24%	84.93%	206	77770	98.56%	89.31%
Portulaca pilosa	1KP	IWIS	IWIS	1091	428545	97.24%	84.89%	207	77827	99.04%	89.37%
Pisonia aculeata	This study		ILU4	1090	452929	97.15%	89.72%	207	79744	99.04%	91.57%
Talinum sp.	1KP	LKKX	LKKX	1090	433206	97.15%	85.81%	206	79708	98.56%	91.53%

Cypselea humifusa	1KP	GJNX	GJNX	1088	407789	96.97%	80.77%	207	73856	99.04%	84.81%
Cucumis sativus	Phytozome		Csat	1086	472685	96.79%	93.63%	206	83204	98.56%	95.55%
Allionia incarnata	1KP	EGOS	EGOS	1086	409817	96.79%	81.18%	206	75493	98.56%	86.69%
Alternanthera tenella	1KP	EYRD (DEMH+ DBCE)	EYRD	1086	410614	96.79%	81.33%	208	74515	99.52%	85.57%
Polycarpaea repens	1KP	RXEN	RXEN	1085	415750	96.70%	82.35%	207	77876	99.04%	89.43%
Alternanthera sessilis	1KP	BWRK (LUNL+ OYST)	BWRK	1083	427908	96.52%	84.76%	205	78355	98.09%	89.98%
Citrus sinensis	Phytozome		Csin	1082	451217	96.43%	89.38%	206	79603	98.56%	91.41%
*Portulaca umbraticola*2	1KP	BLWH (BYNZ+ KDCH)	BLWH	1080	402898	96.26%	79.81%	207	72577	99.04%	83.34%
Sesuvium portulacastrum	1KP	HZTS	HZTS	1080	387038	96.26%	76.66%	205	71340	98.09%	81.92%
Bassia scoparia (syn. *Kochia scoparia*)	1KP	WGET	WGET	1079	402667	96.17%	79.76%	206	76662	98.56%	88.03%
Zaleya pentandra	1KP	BERS	BERS	1078	414246	96.08%	82.05%	206	76078	98.56%	87.36%
Chenopodium quinoa	1KP	SMMC	SMMC	1077	365542	95.99%	72.41%	207	64881	99.04%	74.51%
Boerhavia burbidgeana	1KP	VJPU (GDYP+ HUGU)	VJPU	1076	418612	95.90%	82.92%	208	76947	99.52%	88.36%
Vitis vinifera	Phytozome		Vvin	1074	445129	95.72%	88.17%	201	76522	96.17%	87.87%
Rivina humilis	This study		ILU2	1073	419262	95.63%	83.05%	209	77099	100.00%	88.54%
Simmondsia chinensis	1KP	CVDF	CVDF	1071	443148	95.45%	87.78%	205	79836	98.09%	91.68%
Boerhavia coccinea	1KP	ZBTA (WHHY+ MBWM)	ZBTA	1071	421817	95.45%	83.55%	206	75821	98.56%	87.07%
Mollugo pentaphylla	1KP	KJAA (BZMI+ HURS)	KJAA	1070	406744	95.37%	80.57%	205	71078	98.09%	81.62%
Portulaca suffrutescens	1KP	GCYL	GCYL	1069	418744	95.28%	82.94%	208	79203	99.52%	90.95%
Aerva lanata	1KP	PDQH (VKOE+ EMIG)	PDQH	1069	426890	95.28%	84.56%	205	78701	98.09%	90.38%
Nepenthes alata	1KP	WQUF	WQUF	1068	434599	95.19%	86.08%	204	77976	97.61%	89.54%
Phytolacca americana	1KP	BKQU	BKQU	1067	411562	95.10%	81.52%	207	77428	99.04%	88.91%
Blutaparon vermiculare	1KP	CUTE	CUTE	1067	429699	95.10%	85.11%	203	77181	97.13%	88.63%
Hilleria latifolia	1KP	SFKQ	SFKQ	1067	408931	95.10%	81.00%	209	77470	100.00%	88.96%
Bougainvillea spectabilis	1KP	JAFJ	JAFJ	1065	379473	94.92%	75.17%	203	69467	97.13%	79.77%
Amaranthus retroflexus	1KP	WMLW	WMLW	1063	386343	94.74%	76.53%	206	73385	98.56%	84.27%
Seguieria aculeata	This study		ILU5	1059	436746	94.39%	86.51%	205	80052	98.09%	91.93%
Trianthema portulacastrum	1KP	OMYK (QNIK+ TNVE)	OMYK	1057	371334	94.21%	73.55%	203	69343	97.13%	79.63%
Spergularia media	1KP	TJES	TJES	1056	390758	94.12%	77.40%	205	74930	98.09%	86.05%
Limonium spectabile	1KP	WOBD	WOBD	1056	424814	94.12%	84.15%	204	76709	97.61%	88.09%
Schiedea membranacea	1KP	OLES	OLES	1054	388332	93.94%	76.92%	204	70515	97.61%	80.98%
Lophophora williamsii	1KP	CPKP	CPKP	1052	395623	93.76%	78.36%	205	74468	98.09%	85.51%
Portulaca cryptopetala	1KP	LLQV (VPCP+ TMMR)	LLQV	1051	368281	93.67%	72.95%	199	67178	95.22%	77.14%
Phytolacca bogotensis	1KP	MRKX	MRKX	1051	382587	93.67%	75.78%	206	74038	98.56%	85.02%
Malus domestica	Phytozome		Mdom	1050	449032	93.58%	88.94%	203	80255	97.13%	92.16%
Aerva javanica	1KP	HDSY (GBCQ+	HDSY	1047	392005	93.32%	77.65%	206	73635	98.56%	84.56%

		MEAH)									
Anulocaulis leiosolenus	This study		ILU1	1045	374227	93.14%	74.13%	203	72343	97.13%	83.07%
Portulaca grandiflora	1KP	CPLT	CPLT	1044	388665	93.05%	76.99%	203	73965	97.13%	84.94%
Carica papaya	Phytozome		Cpap	1043	406671	92.96%	80.55%	199	71809	95.22%	82.46%
Portulaca umbraticola	1KP	KDCH	KDCH	1043	379704	92.96%	75.21%	202	69972	96.65%	80.35%
Petiveria alliacea	1KP	AZBL	AZBL	1042	374470	92.87%	74.17%	203	72186	97.13%	82.89%
Portulaca oleracea	1KP	EZGR (TRLB+ CZJT)	EZGR	1042	370530	92.87%	73.39%	201	66595	96.17%	76.47%
Physena madagascariensis	1KP	RUUB	RUUB	1042	359652	92.87%	71.24%	204	72086	97.61%	82.78%
Fragaria vesca	Phytozome		Fves	1038	446494	92.51%	88.44%	200	79041	95.69%	90.77%
Microtea debilis	1KP	YNFJ	YNFJ	1038	338570	92.51%	67.06%	197	64150	94.26%	73.67%
Silene latifolia	1KP	FZQN	FZQN	1037	374873	92.42%	74.25%	199	70778	95.22%	81.28%
Sarcobatus vermiculatus	1KP	GIWN	GIWN	1037	381175	92.42%	75.50%	201	71225	96.17%	81.79%
Portulaca molokiniensis	1KP	UQCB	UQCB	1035	356610	92.25%	70.64%	201	67043	96.17%	76.99%
Mirabilis jalapa	1KP	JGAB (GQAV+ DZRA)	JGAB	1032	380248	91.98%	75.32%	199	69638	95.22%	79.97%
Alternanthera caracasana	1KP	OHKC (WGUG+ VUIT)	OHKC	1022	317674	91.09%	62.92%	204	61109	97.61%	70.17%
Delosperma echinatum	1KP	BJKT	BJKT	1021	337820	91.00%	66.91%	200	69986	95.69%	80.37%
Fallopia convolvulus (*syn. Polygonum convolvulus*)	1KP	FYSJ	FYSJ	1018	356146	90.73%	70.54%	204	70921	97.61%	81.44%
Alternanthera brasiliana	1KP	ZBPY (KTQI+ ENCD)	ZBPY	1014	325422	90.37%	64.46%	197	61145	94.26%	70.22%
Basella alba	1KP	CTYH	CTYH	1012	299934	90.20%	59.41%	200	59788	95.69%	68.66%
Beta maritima	1KP	FVXD	FVXD	1002	294280	89.30%	58.29%	202	62443	96.65%	71.71%
Mollugo cerviana	1KP	RNBN	RNBN	992	394866	88.41%	78.21%	199	73880	95.22%	84.84%
Frankenia laevis	1KP	WPYJ	WPYJ	989	325331	88.15%	64.44%	201	64403	96.17%	73.96%
Solanum tuberosum	Phytozome		Stub	978	395414	87.17%	78.32%	193	74505	92.34%	85.56%
Mollugo verticillata	1KP	NXTS (OFWX+ QASA)	NXTS	905	283078	80.66%	56.07%	168	50685	80.38%	58.20%
Mollugo nudicaulis	1KP	SCAO (CUVY+ UNSW)	SCAO	884	253407	78.79%	50.19%	181	47701	86.60%	54.78%
Saponaria officinalis	1KP	SKNL	SKNL	884	246374	78.79%	48.80%	190	54458	90.91%	62.54%
Medicago truncatula	Phytozome		Mtru	817	352124	72.82%	69.75%	158	65048	75.60%	74.70%
Dianthus caryophyllus	1KP	SHEZ	SHEZ	599	141364	53.39%	28.00%	162	36681	77.51%	42.12%
Beta vulgaris	EST		BVGI	595	113312	53.03%	22.44%	142	26955	67.94%	30.95%
Pereskia aculeata	1KP	JLOV	JLOV	303	64633	27.01%	12.80%	97	20116	46.41%	23.10%
Plumbago auriculata	1KP	CGGO	CGGO	140	26012	12.48%	5.15%	40	8016	19.14%	9.21%

Table S2. Enriched GO terms among homologs containing at least 60 of the 69 Caryophyllales taxa, as found using GOrilla (Eden et al. 2009) with control for False Discovery Rate (FDR; Benjamini and Hochberg 1995).

GO categories	GO term	Description	P-value	FDR q-value
Process	GO:0044281	small molecule metabolic process	1.17E-16	2.41E-13
Process	GO:1901564	organonitrogen compound metabolic process	1.42E-15	1.48E-12
Process	GO:0044711	single-organism biosynthetic process	4.12E-15	2.84E-12
Process	GO:0006520	cellular amino acid metabolic process	2.83E-14	1.47E-11
Process	GO:0043436	oxoacid metabolic process	8.71E-14	3.61E-11
Process	GO:0006082	organic acid metabolic process	8.71E-14	3.01E-11
Process	GO:0019752	carboxylic acid metabolic process	8.71E-14	2.58E-11
Process	GO:0044699	single-organism process	5.9E-13	1.53E-10
Process	GO:0044723	single-organism carbohydrate metabolic process	1.82E-12	4.2E-10
Process	GO:0008152	metabolic process	7.57E-12	1.57E-9
Process	GO:1901566	organonitrogen compound biosynthetic process	1.49E-11	2.8E-9
Process	GO:0009056	catabolic process	1.99E-11	3.44E-9
Process	GO:0005975	carbohydrate metabolic process	4.52E-11	7.21E-9
Process	GO:1901575	organic substance catabolic process	4.59E-11	6.79E-9
Process	GO:0044710	single-organism metabolic process	5.87E-11	8.1E-9
Process	GO:0071704	organic substance metabolic process	7.55E-11	9.77E-9
Process	GO:0016192	vesicle-mediated transport	3.57E-10	4.35E-8
Process	GO:0051234	establishment of localization	4.33E-10	4.99E-8
Process	GO:0008652	cellular amino acid biosynthetic process	7.59E-10	8.28E-8
Process	GO:0044237	cellular metabolic process	1.43E-9	1.48E-7
Process	GO:0006810	transport	3.17E-9	3.13E-7
Process	GO:0051649	establishment of localization in cell	4.42E-9	4.16E-7
Process	GO:0016051	carbohydrate biosynthetic process	5.25E-9	4.73E-7
Process	GO:0044238	primary metabolic process	5.44E-9	4.69E-7
Process	GO:1901362	organic cyclic compound biosynthetic process	5.73E-9	4.75E-7
Process	GO:0009987	cellular process	8.88E-9	7.08E-7
Process	GO:0019438	aromatic compound biosynthetic process	1.15E-8	8.84E-7
Process	GO:0005996	monosaccharide metabolic process	1.53E-8	1.13E-6
Process	GO:1901607	alpha-amino acid biosynthetic process	2.58E-8	1.84E-6
Process	GO:0006807	nitrogen compound metabolic process	3E-8	2.07E-6
Process	GO:0044765	single-organism transport	3.09E-8	2.06E-6
Process	GO:0048193	Golgi vesicle transport	3.2E-8	2.07E-6
Process	GO:0010038	response to metal ion	3.23E-8	2.03E-6
Process	GO:0046907	intracellular transport	5.14E-8	3.13E-6
Process	GO:1901605	alpha-amino acid metabolic process	6E-8	3.55E-6
Process	GO:0009058	biosynthetic process	7.38E-8	4.25E-6
Process	GO:0019318	hexose metabolic process	8.28E-8	4.63E-6
Process	GO:0044248	cellular catabolic process	9.33E-8	5.08E-6
Process	GO:0044283	small molecule biosynthetic process	1.06E-7	5.65E-6
Process	GO:1901135	carbohydrate derivative metabolic process	1.44E-7	7.47E-6
Process	GO:1902582	single-organism intracellular transport	1.52E-7	7.68E-6
Process	GO:0044712	single-organism catabolic process	1.93E-7	9.53E-6
Process	GO:0044272	sulfur compound biosynthetic process	1.95E-7	9.4E-6
Process	GO:0044763	single-organism cellular process	2E-7	9.4E-6
Process	GO:0044271	cellular nitrogen compound biosynthetic process	2.11E-7	9.71E-6
Process	GO:0071702	organic substance transport	2.23E-7	1E-5
Process	GO:0016053	organic acid biosynthetic process	2.27E-7	1E-5
Process	GO:0046394	carboxylic acid biosynthetic process	2.27E-7	9.8E-6
Process	GO:0006790	sulfur compound metabolic process	2.57E-7	1.08E-5

Process	GO:1901360	organic cyclic compound metabolic process	2.6E-7	1.08E-5
Process	GO:0044262	cellular carbohydrate metabolic process	2.71E-7	1.1E-5
Process	GO:0006753	nucleoside phosphate metabolic process	2.96E-7	1.18E-5
Process	GO:0018130	heterocycle biosynthetic process	3.11E-7	1.22E-5
Process	GO:0019637	organophosphate metabolic process	3.49E-7	1.34E-5
Process	GO:0046686	response to cadmium ion	3.63E-7	1.37E-5
Process	GO:0006418	tRNA aminoacylation for protein translation	3.86E-7	1.43E-5
Process	GO:0043038	amino acid activation	3.86E-7	1.4E-5
Process	GO:0043039	tRNA aminoacylation	3.86E-7	1.38E-5
Process	GO:1901576	organic substance biosynthetic process	4.34E-7	1.52E-5
Process	GO:0009117	nucleotide metabolic process	4.5E-7	1.55E-5
Process	GO:0006508	proteolysis	5.11E-7	1.73E-5
Process	GO:0090407	organophosphate biosynthetic process	6E-7	2.01E-5
Process	GO:0006725	cellular aromatic compound metabolic process	6.72E-7	2.21E-5
Process	GO:0016485	protein processing	8.2E-7	2.65E-5
Process	GO:0006006	glucose metabolic process	8.63E-7	2.75E-5
Process	GO:0034654	nucleobase-containing compound biosynthetic process	1.03E-6	3.22E-5
Process	GO:0051604	protein maturation	1.17E-6	3.6E-5
Process	GO:1901293	nucleoside phosphate biosynthetic process	1.18E-6	3.6E-5
Process	GO:0000096	sulfur amino acid metabolic process	1.67E-6	5.02E-5
Process	GO:0055086	nucleobase-containing small molecule metabolic process	1.7E-6	5.02E-5
Process	GO:0009165	nucleotide biosynthetic process	1.91E-6	5.57E-5
Process	GO:0009057	macromolecule catabolic process	2.43E-6	7E-5
Process	GO:0016482	cytoplasmic transport	2.67E-6	7.57E-5
Process	GO:0016070	RNA metabolic process	4.45E-6	1.25E-4
Process	GO:0032787	monocarboxylic acid metabolic process	5.71E-6	1.58E-4
Process	GO:0009250	glucan biosynthetic process	5.8E-6	1.58E-4
Process	GO:0006090	pyruvate metabolic process	7.21E-6	1.94E-4
Process	GO:0046483	heterocycle metabolic process	8.5E-6	2.26E-4
Process	GO:0000097	sulfur amino acid biosynthetic process	8.69E-6	2.28E-4
Process	GO:0019682	glyceraldehyde-3-phosphate metabolic process	1.18E-5	3.05E-4
Process	GO:0019288	isopentenyl diphosphate biosynthetic process, methylerythritol 4-phosphate pathway	1.18E-5	3.01E-4
Process	GO:0009240	isopentenyl diphosphate biosynthetic process	1.18E-5	2.98E-4
Process	GO:0046490	isopentenyl diphosphate metabolic process	1.18E-5	2.94E-4
Process	GO:0044249	cellular biosynthetic process	1.2E-5	2.96E-4
Process	GO:0010498	proteasomal protein catabolic process	1.3E-5	3.17E-4
Process	GO:0006073	cellular glucan metabolic process	1.37E-5	3.3E-4
Process	GO:0044042	glucan metabolic process	1.37E-5	3.27E-4
Process	GO:0019252	starch biosynthetic process	1.49E-5	3.5E-4
Process	GO:0044724	single-organism carbohydrate catabolic process	1.51E-5	3.51E-4
Process	GO:0051603	proteolysis involved in cellular protein catabolic process	1.6E-5	3.68E-4
Process	GO:0005982	starch metabolic process	1.92E-5	4.37E-4
Process	GO:0009311	oligosaccharide metabolic process	1.92E-5	4.32E-4
Process	GO:0034637	cellular carbohydrate biosynthetic process	2.2E-5	4.9E-4
Process	GO:0009072	aromatic amino acid family metabolic process	2.35E-5	5.18E-4
Process	GO:0009069	serine family amino acid metabolic process	2.44E-5	5.32E-4
Process	GO:0005984	disaccharide metabolic process	2.52E-5	5.44E-4
Process	GO:0016052	carbohydrate catabolic process	2.59E-5	5.52E-4
Process	GO:0009651	response to salt stress	3.16E-5	6.67E-4
Process	GO:0000271	polysaccharide biosynthetic process	3.29E-5	6.89E-4
Process	GO:0006970	response to osmotic stress	3.3E-5	6.83E-4
Process	GO:0015031	protein transport	3.32E-5	6.8E-4
Process	GO:0045184	establishment of protein localization	3.32E-5	6.74E-4

Process	GO:0006094	gluconeogenesis	3.38E-5	6.8E-4
Process	GO:0019319	hexose biosynthetic process	3.38E-5	6.74E-4
Process	GO:0005976	polysaccharide metabolic process	3.4E-5	6.7E-4
Process	GO:0034641	cellular nitrogen compound metabolic process	3.82E-5	7.45E-4
Process	GO:0030163	protein catabolic process	4.07E-5	7.88E-4
Process	GO:0006605	protein targeting	4.45E-5	8.54E-4
Process	GO:0046364	monosaccharide biosynthetic process	4.54E-5	8.63E-4
Process	GO:0044265	cellular macromolecule catabolic process	5.59E-5	1.05E-3
Process	GO:0000023	maltose metabolic process	6.32E-5	1.18E-3
Process	GO:0006081	cellular aldehyde metabolic process	6.47E-5	1.2E-3
Process	GO:1901137	carbohydrate derivative biosynthetic process	6.56E-5	1.2E-3
Process	GO:0033692	cellular polysaccharide biosynthetic process	6.86E-5	1.25E-3
Process	GO:0006793	phosphorus metabolic process	8.14E-5	1.47E-3
Process	GO:0006886	intracellular protein transport	8.21E-5	1.47E-3
Process	GO:0072527	pyrimidine-containing compound metabolic process	9.47E-5	1.68E-3
Process	GO:0072528	pyrimidine-containing compound biosynthetic process	9.47E-5	1.66E-3
Process	GO:0044264	cellular polysaccharide metabolic process	9.92E-5	1.73E-3
Process	GO:0051186	cofactor metabolic process	9.96E-5	1.72E-3
Process	GO:0042440	pigment metabolic process	1.02E-4	1.75E-3
Process	GO:0046390	ribose phosphate biosynthetic process	1.03E-4	1.74E-3
Process	GO:0009260	ribonucleotide biosynthetic process	1.03E-4	1.73E-3
Process	GO:0072594	establishment of protein localization to organelle	1.09E-4	1.82E-3
Process	GO:0043170	macromolecule metabolic process	1.16E-4	1.93E-3
Process	GO:0006498	N-terminal protein lipidation	1.19E-4	1.96E-3
Process	GO:0006499	N-terminal protein myristoylation	1.19E-4	1.94E-3
Process	GO:0006623	protein targeting to vacuole	1.19E-4	1.93E-3
Process	GO:0018377	protein myristoylation	1.19E-4	1.91E-3
Process	GO:0072666	establishment of protein localization to vacuole	1.19E-4	1.9E-3
Process	GO:0009070	serine family amino acid biosynthetic process	1.19E-4	1.88E-3
Process	GO:0031365	N-terminal protein amino acid modification	1.19E-4	1.87E-3
Process	GO:0050896	response to stimulus	1.29E-4	2.01E-3
Process	GO:0006221	pyrimidine nucleotide biosynthetic process	1.53E-4	2.37E-3
Process	GO:0006220	pyrimidine nucleotide metabolic process	1.53E-4	2.35E-3
Process	GO:0009259	ribonucleotide metabolic process	1.75E-4	2.67E-3
Process	GO:0019693	ribose phosphate metabolic process	1.75E-4	2.65E-3
Process	GO:0006399	tRNA metabolic process	1.82E-4	2.73E-3
Process	GO:0006139	nucleobase-containing compound metabolic process	1.88E-4	2.8E-3
Process	GO:0006534	cysteine metabolic process	2.2E-4	3.26E-3
Process	GO:0009220	pyrimidine ribonucleotide biosynthetic process	2.45E-4	3.6E-3
Process	GO:0009218	pyrimidine ribonucleotide metabolic process	2.45E-4	3.58E-3
Process	GO:0010035	response to inorganic substance	2.45E-4	3.55E-3
Process	GO:0043543	protein acylation	2.78E-4	4E-3
Process	GO:0006096	glycolytic process	2.78E-4	3.97E-3
Process	GO:1901361	organic cyclic compound catabolic process	3.62E-4	5.14E-3
Process	GO:0046700	heterocycle catabolic process	3.62E-4	5.1E-3
Process	GO:0009658	chloroplast organization	3.9E-4	5.45E-3
Process	GO:0015994	chlorophyll metabolic process	3.94E-4	5.48E-3
Process	GO:0019344	cysteine biosynthetic process	4.04E-4	5.57E-3
Process	GO:0016071	mRNA metabolic process	4.17E-4	5.72E-3
Process	GO:0006796	phosphate-containing compound metabolic process	4.33E-4	5.9E-3
Process	GO:0009628	response to abiotic stimulus	4.67E-4	6.32E-3
Process	GO:0050790	regulation of catalytic activity	5.11E-4	6.88E-3
Process	GO:0042221	response to chemical	5.22E-4	6.97E-3
Process	GO:0019439	aromatic compound catabolic process	5.44E-4	7.23E-3

Process	GO:0044270	cellular nitrogen compound catabolic process	5.44E-4	7.18E-3
Process	GO:0009073	aromatic amino acid family biosynthetic process	5.83E-4	7.64E-3
Process	GO:0044275	cellular carbohydrate catabolic process	6.95E-4	9.05E-3
Process	GO:0009067	aspartate family amino acid biosynthetic process	6.95E-4	8.99E-3
Process	GO:0019320	hexose catabolic process	7.64E-4	9.83E-3
Process	GO:0046365	monosaccharide catabolic process	7.64E-4	9.77E-3
Process	GO:0034660	ncRNA metabolic process	8.28E-4	1.05E-2
Process	GO:0043632	modification-dependent macromolecule catabolic process	8.69E-4	1.1E-2
Process	GO:0006511	ubiquitin-dependent protein catabolic process	8.69E-4	1.09E-2
Process	GO:0019941	modification-dependent protein catabolic process	8.69E-4	1.08E-2
Function	GO:0004812	aminoacyl-tRNA ligase activity	6.77E-8	6.22E-5
Function	GO:0016875	ligase activity, forming carbon-oxygen bonds	6.77E-8	3.11E-5
Function	GO:0016876	ligase activity, forming aminoacyl-tRNA and related compounds	6.77E-8	2.07E-5
Function	GO:0003824	catalytic activity	1.6E-6	3.69E-4
Function	GO:0016874	ligase activity	1.67E-6	3.07E-4
Function	GO:0003723	RNA binding	6.69E-5	1.03E-2
Function	GO:1901363	heterocyclic compound binding	2.47E-4	3.24E-2
Function	GO:0097159	organic cyclic compound binding	2.47E-4	2.83E-2
Function	GO:0005488	binding	2.54E-4	2.59E-2
Function	GO:0003743	translation initiation factor activity	6.08E-4	5.59E-2
Function	GO:0036094	small molecule binding	9.48E-4	7.92E-2
Component	GO:0005829	cytosol	5.55E-17	1.39E-14
Component	GO:0044434	chloroplast part	1.45E-12	1.82E-10
Component	GO:0044435	plastid part	4.73E-12	3.96E-10
Component	GO:0009570	chloroplast stroma	9.08E-12	5.7E-10
Component	GO:0009532	plastid stroma	9.08E-12	4.56E-10
Component	GO:0044444	cytoplasmic part	1.45E-8	6.09E-7
Component	GO:0044446	intracellular organelle part	4.52E-8	1.62E-6
Component	GO:0009507	chloroplast	5.47E-8	1.72E-6
Component	GO:0044422	organelle part	5.58E-8	1.56E-6
Component	GO:0009536	plastid	8.74E-8	2.19E-6
Component	GO:0005737	cytoplasm	2.82E-7	6.42E-6
Component	GO:0044464	cell part	3.46E-7	7.23E-6
Component	GO:0009941	chloroplast envelope	2.43E-6	4.7E-5
Component	GO:0009526	plastid envelope	3.64E-6	6.53E-5
Component	GO:0016020	membrane	8.39E-6	1.4E-4
Component	GO:0044424	intracellular part	1.08E-5	1.7E-4
Component	GO:0031975	envelope	1.2E-4	1.77E-3
Component	GO:0031967	organelle envelope	1.2E-4	1.67E-3
Component	GO:0005777	peroxisome	1.67E-4	2.21E-3
Component	GO:0031090	organelle membrane	2.43E-4	3.05E-3
Component	GO:0005802	trans-Golgi network	2.61E-4	3.12E-3
Component	GO:0005794	Golgi apparatus	2.73E-4	3.11E-3
Component	GO:0005768	endosome	2.77E-4	3.02E-3
Component	GO:0005886	plasma membrane	4.32E-4	4.52E-3
Component	GO:0098588	bounding membrane of organelle	5.89E-4	5.91E-3
Component	GO:0005783	endoplasmic reticulum	8.03E-4	7.75E-3

Table S3. Collection information and SRA accession numbers for the six newly generated transcriptome data sets. Taxon codes are the shortened taxon names used throughout the analyses. OC = Oberlin College herbarium.

Taxon code	SRA accession	# Reads	Collection # (Herbarium)	Taxon name & authority	RNA	Collection Locality
ILU1	SRX 717838	28,807,692	M. J. Moore 1070 (OC)	*Anulocaulis leiosolenus* (Torr.) Standl. var. *gypsogenus* (Waterf.) Spellenb. & T.Wootten	RNA isolated from one flower bud on 16 Mar 2012 by Moore; tissue taken for RNA isolation was from the same individual as voucher specimen	USA, Texas, Culberson County: Along FM 652 25 mi W of jct w/ US 285 in Orla. 31 53 04.1 N, 104 19 14.7 W. Elevation: 3491 ft. Collected in liquid nitrogen September 24, 2010
ILU2	SRX 718277	28,241,891	M. J. Moore 1651 (OC)	*Rivina humilis* L.	RNA from inflorescence + young leaf on 23 Mar 2012 by Moore	Cultivated at home of Dr. Norm Douglas. Tissue collected 23 Mar 2012
ILU3	SRX 718384	35,112,814	Kew Living Collection #2011-994	*Guapira obtusata* (Jacq.) Little	RNA from young leaves and meristem. Tissue collected and RNA isolated by Brockington March 2012	Cultivated at Royal Botanic Gardens, Kew.
ILU4	SRX 718389	34,242,340	Kew Living Collection #2011-448.	*Pisonia aculeata* L.	RNA from young leaves and meristem. Tissue collected and RNA isolated by Brockington March 2012	Cultivated at Royal Botanic Gardens, Kew.
ILU5	SRX 718486	30,654,039	Kew Living Collection #1991-169.	*Seguieria aculeata* Jacq.	RNA from young leaves and meristem. Tissue collected and RNA isolated by Brockington March 2012	Cultivated at Royal Botanic Gardens, Kew.
ILU6	SRX 718672	31,855,870	Kew Living Collection #1986-4920	*Bougainvillea stipitata* Griseb. var. *grisebachiana* Heimerl	RNA from young leaves and meristem. Tissue collected and RNA isolated by Brockington March 2012	Cultivated at Royal Botanic Gardens, Kew.

Figure S1. Species trees from (a) the 1,122-gene data set and (b) the 209-gene data set. Support values not shown if all are 100%, and each "*" indicates the corresponding support value being 100%. Caryophyllales branches that are relatively unstable topologically are marked with arrows.

Figure S2. Distribution of synonymous substitutions (Ks) for Caryophyllales paralogs. The diameter of the circles on nodes is proportional to the percentage of genes showing duplications at that node. Horizontal arrows point to branches with relatively low support values for the species tree. Squares on branches indicate genome duplications supported by peaks in Ks plots.

Figure S3. Phylogenetic tree for the homolog group containing the *Beta vulgaris CYP76AD1*. Gene sequences that have been experimentally characterized to encode a key enzyme in the betalain synthesis pathway were downloaded from GenBank, added to the alignment and highlighted in the resulting phylogenetic tree (Hatlestad *et al.* 2012). Sequence IDs are coded red for taxa that fall into the phytolaccoid clade in the species tree (Fig. 3), blue for taxa fall into Portulacineae and Molluginaceae, and green for taxa of Amaranthaceae and Caryophyllaceae.

References

Benjamini Y, Hochberg Y. 1995. Controlling the false discovery rate: a practical and powerful approach to multiple testing. *Journal of the Royal Statistical Society. Series B (Methodological)* 57(1):289–300.

Eden E, Navon R, Steinfeld I, Lipson D, Yakhini Z. 2009. GOrilla: a tool for discovery and visualization of enriched GO terms in ranked gene lists. *BMC Bioinformatics* 10(1):48.

Hatlestad GJ, Sunnadeniya RM, Akhavan NA, Gonzalez A, Goldman IL, McGrath JM, Lloyd AM. 2012. The beet R locus encodes a new cytochrome P450 required for red betalain production. *Nat Genet* 44(7):816–820.

Fig. S1

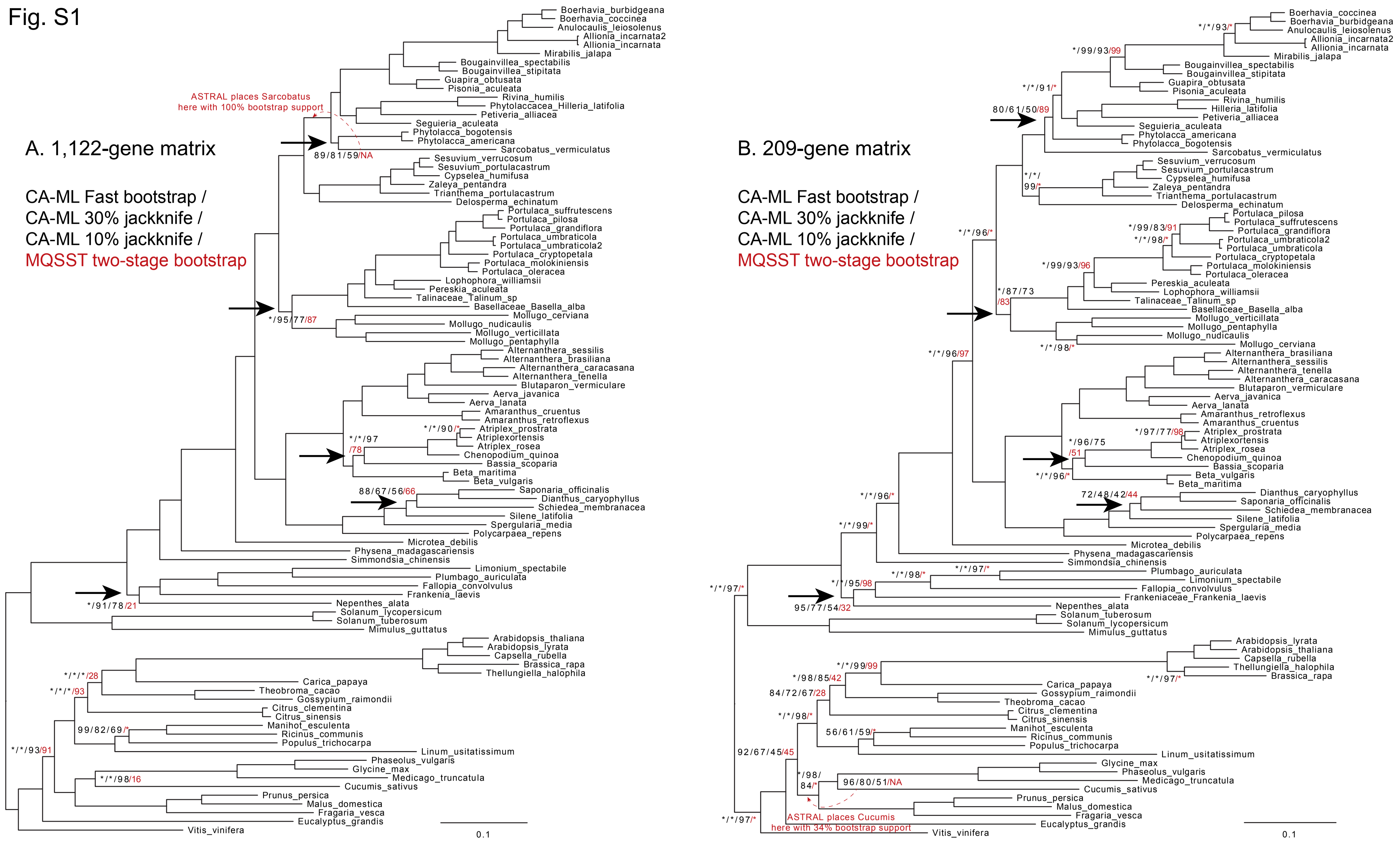
A. 1,122-gene matrix
CA-ML Fast bootstrap /
CA-ML 30% jackknife /
CA-ML 10% jackknife /
MQSST two-stage bootstrap
ASTRAL places Sarcobatus
here with 100% bootstrap support
89/81/59/NA
*/95/77/87
//97
/78
//90/*
88/67/56/66
*/91/78/21
//*/28
//*/93
99/82/69/*
//93/91
//98/16
Boerhavia_burbidgeana
Boerhavia_coccinea
Anulocaulis_leiosolenus
Allionia_incarnata2
Allionia_incarnata
Mirabilis_jalapa
Bougainvillea_spectabilis
Bougainvillea_stipitata
Guapira_obtusata
Pisonia_aculeata
Rivina_humilis
Phytolaccacea_Hilleria_latifolia
Petiveria_alliacea
Seguieria_aculeata
Phytolacca_bogotensis
Phytolacca_americana
Sarcobatus_vermiculatus
Sesuvium_verrucosum
Sesuvium_portulacastrum
Cypselea_humifusa
Zaleya_pentandra
Trianthema_portulacastrum
Delosperma_echinatum
Portulaca_suffrutescens
Portulaca_pilosa
Portulaca_grandiflora
Portulaca_umbraticola
Portulaca_umbraticola2
Portulaca_cryptopetala
Portulaca_molokiniensis
Portulaca_oleracea
Lophophora_williamsii
Pereskia_aculeata
Talinaceae_Talinum_sp
Basellaceae_Basella_alba
Mollugo_cerviana
Mollugo_nudicaulis
Mollugo_verticillata
Mollugo_pentaphylla
Alternanthera_sessilis
Alternanthera_brasiliana
Alternanthera_caracasana
Alternanthera_tenella
Blutaparon_vermiculare
Aerva_javanica
Aerva_lanata
Amaranthus_cruentus
Amaranthus_retroflexus
Atriplex_prostrata
Atriplexortensis
Atriplex_rosea
Chenopodium_quinoa
Bassia_scoparia
Beta_maritima
Beta_vulgaris
Saponaria_officinalis
Dianthus_caryophyllus
Schiedea_membranacea
Silene_latifolia
Spergularia_media
Polycarpaea_repens
Microtea_debilis
Physena_madagascariensis
Simmondsia_chinensis
Limonium_spectabile
Plumbago_auriculata
Fallopia_convolvulus
Frankenia_laevis
Nepenthes_alata
Solanum_lycopersicum
Solanum_tuberosum
Mimulus_guttatus
Arabidopsis_thaliana
Arabidopsis_lyrata
Capsella_rubella
Brassica_rapa
Thellungiella_halophila
Carica_papaya
Theobroma_cacao
Gossypium_raimondii
Citrus_clementina
Citrus_sinensis
Manihot_esculenta
Ricinus_communis
Populus_trichocarpa
Linum_usitatissimum
Phaseolus_vulgaris
Glycine_max
Medicago_truncatula
Cucumis_sativus
Prunus_persica
Malus_domestica
Fragaria_vesca
Eucalyptus_grandis
Vitis_vinifera
0.1
B. 209-gene matrix
CA-ML Fast bootstrap /
CA-ML 30% jackknife /
CA-ML 10% jackknife /
MQSST two-stage bootstrap
//93/*
*/99/93/99
//91/*
80/61/50/89
//
99/*
//96/*
*/99/83/91
//98/*
*/99/93/96
*/87/73
/83
//98/*
//96/97
*/97/77/98
*/96/75
/51
//96/*
72/48/42/44
//96/*
//99/*
//98/*
//97/*
//95/98
95/77/54/32
//97/*
//99/99
*/98/85/42
84/72/67/28
//98/*
56/61/59/*
92/67/45/45
*/98/
84/*
96/80/51/NA
//97/*
ASTRAL places Cucumis
here with 34% bootstrap support
Boerhavia_coccinea
Boerhavia_burbidgeana
Anulocaulis_leiosolenus
Allionia_incarnata2
Allionia_incarnata
Mirabilis_jalapa
Bougainvillea_spectabilis
Bougainvillea_stipitata
Guapira_obtusata
Pisonia_aculeata
Rivina_humilis
Hilleria_latifolia
Petiveria_alliacea
Seguieria_aculeata
Phytolacca_americana
Phytolacca_bogotensis
Sarcobatus_vermiculatus
Sesuvium_verrucosum
Sesuvium_portulacastrum
Cypselea_humifusa
Zaleya_pentandra
Trianthema_portulacastrum
Delosperma_echinatum
Portulaca_pilosa
Portulaca_suffrutescens
Portulaca_grandiflora
Portulaca_umbraticola2
Portulaca_umbraticola
Portulaca_cryptopetala
Portulaca_molokiniensis
Portulaca_oleracea
Pereskia_aculeata
Lophophora_williamsii
Talinaceae_Talinum_sp
Basellaceae_Basella_alba
Mollugo_verticillata
Mollugo_pentaphylla
Mollugo_nudicaulis
Mollugo_cerviana
Alternanthera_brasiliana
Alternanthera_sessilis
Alternanthera_tenella
Alternanthera_caracasana
Blutaparon_vermiculare
Aerva_javanica
Aerva_lanata
Amaranthus_retroflexus
Amaranthus_cruentus
Atriplex_prostrata
Atriplexortensis
Atriplex_rosea
Chenopodium_quinoa
Bassia_scoparia
Beta_vulgaris
Beta_maritima
Dianthus_caryophyllus
Saponaria_officinalis
Schiedea_membranacea
Silene_latifolia
Spergularia_media
Polycarpaea_repens
Microtea_debilis
Physena_madagascariensis
Simmondsia_chinensis
Plumbago_auriculata
Limonium_spectabile
Fallopia_convolvulus
Frankeniaceae_Frankenia_laevis
Nepenthes_alata
Solanum_tuberosum
Solanum_lycopersicum
Mimulus_guttatus
Arabidopsis_lyrata
Arabidopsis_thaliana
Capsella_rubella
Thellungiella_halophila
Brassica_rapa
Carica_papaya
Gossypium_raimondii
Theobroma_cacao
Citrus_clementina
Citrus_sinensis
Manihot_esculenta
Ricinus_communis
Populus_trichocarpa
Linum_usitatissimum
Glycine_max
Phaseolus_vulgaris
Medicago_truncatula
Cucumis_sativus
Prunus_persica
Malus_domestica
Fragaria_vesca
Eucalyptus_grandis
Vitis_vinifera
0.1

Figure S2

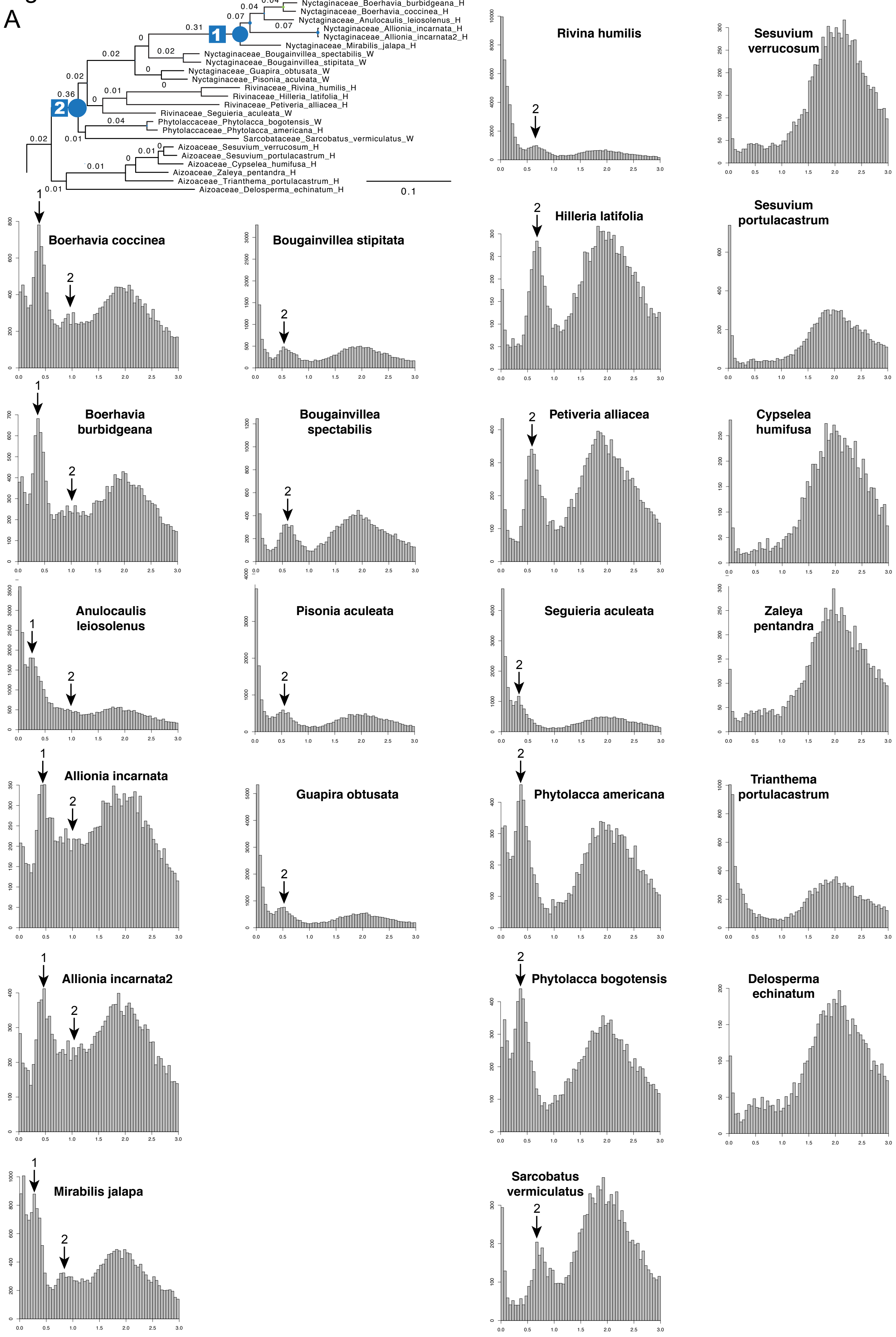
A
Nyctaginaceae_Boerhavia_burbidgeana_H
Nyctaginaceae_Boerhavia_coccinea_H
Nyctaginaceae_Anulocaulis_leiosolenus_H
Nyctaginaceae_Allionia_incarnata_H
Nyctaginaceae_Allionia_incarnata2_H
Nyctaginaceae_Mirabilis_jalapa_H
Nyctaginaceae_Bougainvillea_spectabilis_W
Nyctaginaceae_Bougainvillea_stipitata_W
Nyctaginaceae_Guapira_obtusata_W
Nyctaginaceae_Pisonia_aculeata_W
Rivinaceae_Rivina_humilis_H
Rivinaceae_Hilleria_latifolia_H
Rivinaceae_Petiveria_alliacea_H
Rivinaceae_Seguieria_aculeata_W
Phytolaccaceae_Phytolacca_bogotensis_W
Phytolaccaceae_Phytolacca_americana_H
Sarcobataceae_Sarcobatus_vermiculatus_W
Aizoaceae_Sesuvium_verrucosum_H
Aizoaceae_Sesuvium_portulacastrum_H
Aizoaceae_Cypselea_humifusa_H
Aizoaceae_Zaleya_pentandra_H
Aizoaceae_Trianthema_portulacastrum_H
Aizoaceae_Delosperma_echinatum_H
0.1
Rivina humilis
Sesuvium verrucosum
Boerhavia coccinea
Bougainvillea stipitata
Hilleria latifolia
Sesuvium portulacastrum
Boerhavia burbidgeana
Bougainvillea spectabilis
Petiveria alliacea
Cypselea humifusa
Anulocaulis leiosolenus
Pisonia aculeata
Seguieria aculeata
Zaleya pentandra
Allionia incarnata
Guapira obtusata
Phytolacca americana
Trianthema portulacastrum
Allionia incarnata2
Phytolacca bogotensis
Delosperma echinatum
Mirabilis jalapa
Sarcobatus vermiculatus

Figure S2
B

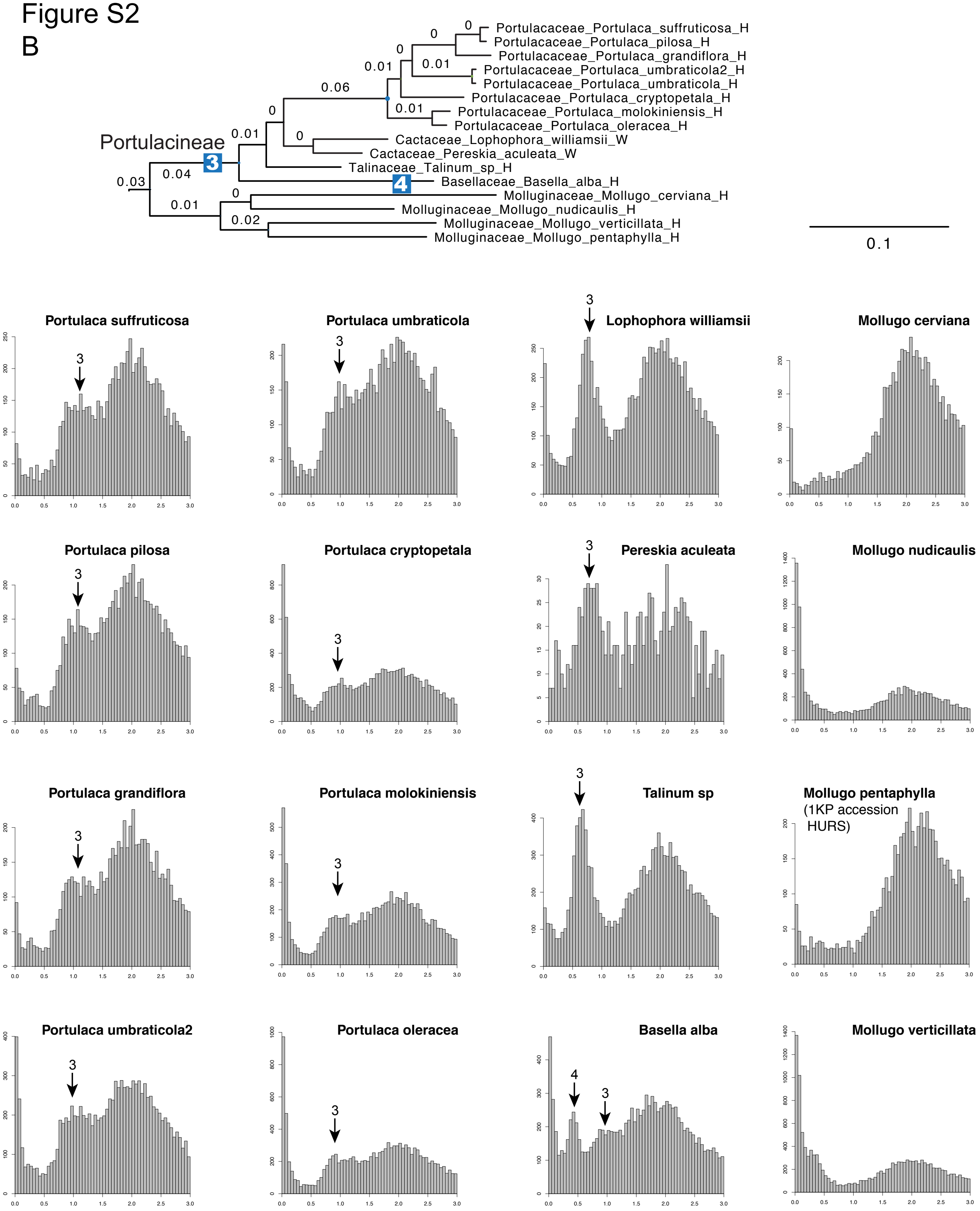

Portulacineae
Portulacaceae_Portulaca_suffruticosa_H
Portulacaceae_Portulaca_pilosa_H
Portulacaceae_Portulaca_grandiflora_H
Portulacaceae_Portulaca_umbraticola2_H
Portulacaceae_Portulaca_umbraticola_H
Portulacaceae_Portulaca_cryptopetala_H
Portulacaceae_Portulaca_molokiniensis_H
Portulacaceae_Portulaca_oleracea_H
Cactaceae_Lophophora_williamsii_W
Cactaceae_Pereskia_aculeata_W
Talinaceae_Talinum_sp_H
Basellaceae_Basella_alba_H
Molluginaceae_Mollugo_cerviana_H
Molluginaceae_Mollugo_nudicaulis_H
Molluginaceae_Mollugo_verticillata_H
Molluginaceae_Mollugo_pentaphylla_H
0.1
Portulaca suffruticosa
Portulaca umbraticola
Lophophora williamsii
Mollugo cerviana
Portulaca pilosa
Portulaca cryptopetala
Pereskia aculeata
Mollugo nudicaulis
Portulaca grandiflora
Portulaca molokiniensis
Talinum sp
Mollugo pentaphylla
(1KP accession
HURS)
Portulaca umbraticola2
Portulaca oleracea
Basella alba
Mollugo verticillata

Figure S2

C

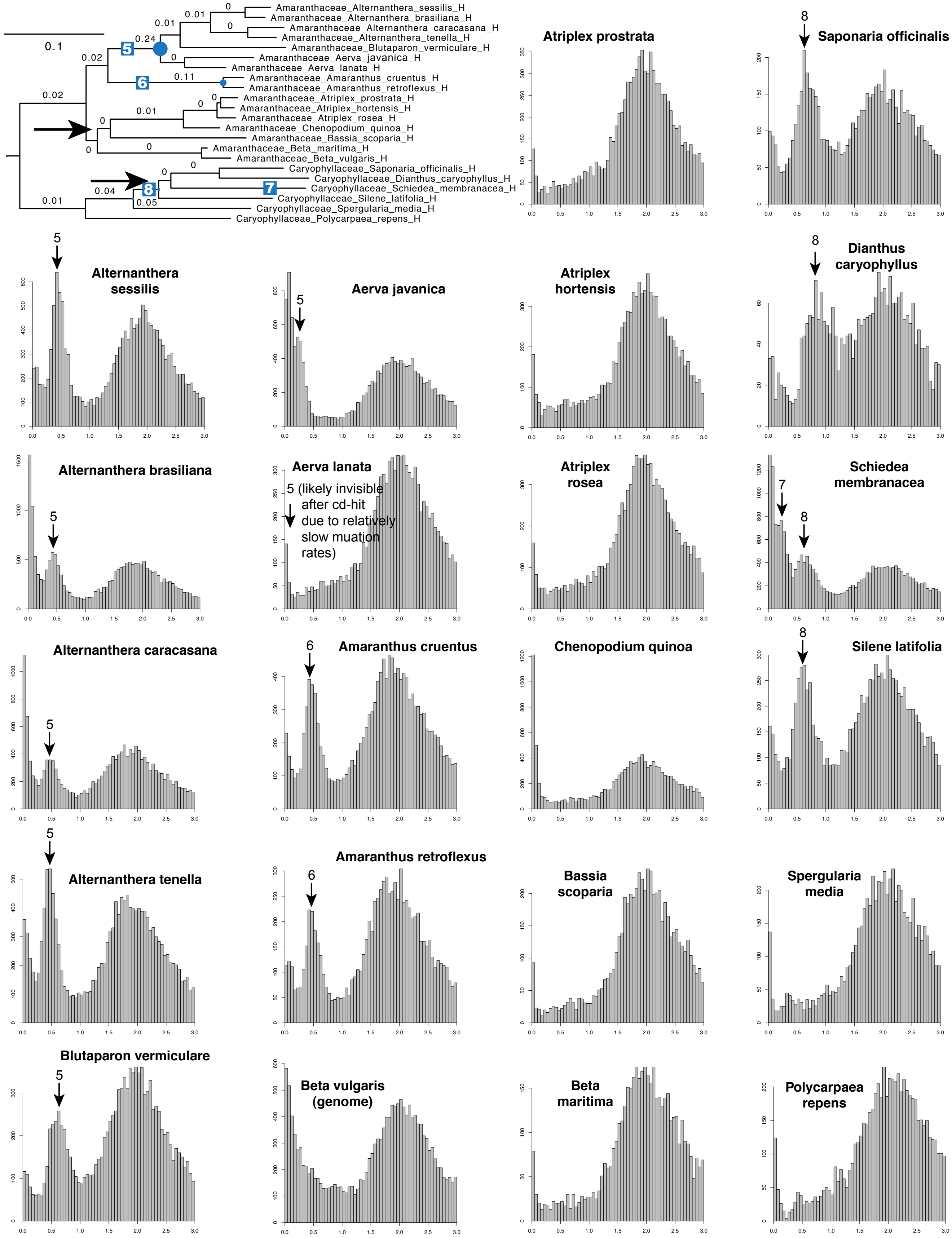
0.1
Amaranthaceae_Alternanthera_sessilis_H
Amaranthaceae_Alternanthera_brasiliana_H
Amaranthaceae_Alternanthera_caracasana_H
Amaranthaceae_Alternanthera_tenella_H
Amaranthaceae_Blutaparon_vermiculare_H
Amaranthaceae_Aerva_javanica_H
Amaranthaceae_Aerva_lanata_H
Amaranthaceae_Amaranthus_cruentus_H
Amaranthaceae_Amaranthus_retroflexus_H
Amaranthaceae_Atriplex_prostrata_H
Amaranthaceae_Atriplex_hortensis_H
Amaranthaceae_Atriplex_rosea_H
Amaranthaceae_Chenopodium_quinoa_H
Amaranthaceae_Bassia_scoparia_H
Amaranthaceae_Beta_maritima_H
Amaranthaceae_Beta_vulgaris_H
Caryophyllaceae_Saponaria_officinalis_H
Caryophyllaceae_Dianthus_caryophyllus_H
Caryophyllaceae_Schiedea_membranacea_H
Caryophyllaceae_Silene_latifolia_H
Caryophyllaceae_Spergularia_media_H
Caryophyllaceae_Polycarpaea_repens_H
0.24
0.11
0.04
0.05
0.02
0.01
5
6
7
8
Atriplex prostrata
Saponaria officinalis
Alternanthera sessilis
Aerva javanica
Atriplex hortensis
Dianthus caryophyllus
Alternanthera brasiliana
Aerva lanata
5 (likely invisible after cd-hit due to relatively slow muation rates)
Atriplex rosea
Schiedea membranacea
Alternanthera caracasana
Amaranthus cruentus
Chenopodium quinoa
Silene latifolia
Alternanthera tenella
Amaranthus retroflexus
Bassia scoparia
Spergularia media
Blutaparon vermiculare
Beta vulgaris (genome)
Beta maritima
Polycarpaea repens

Figure S2
D

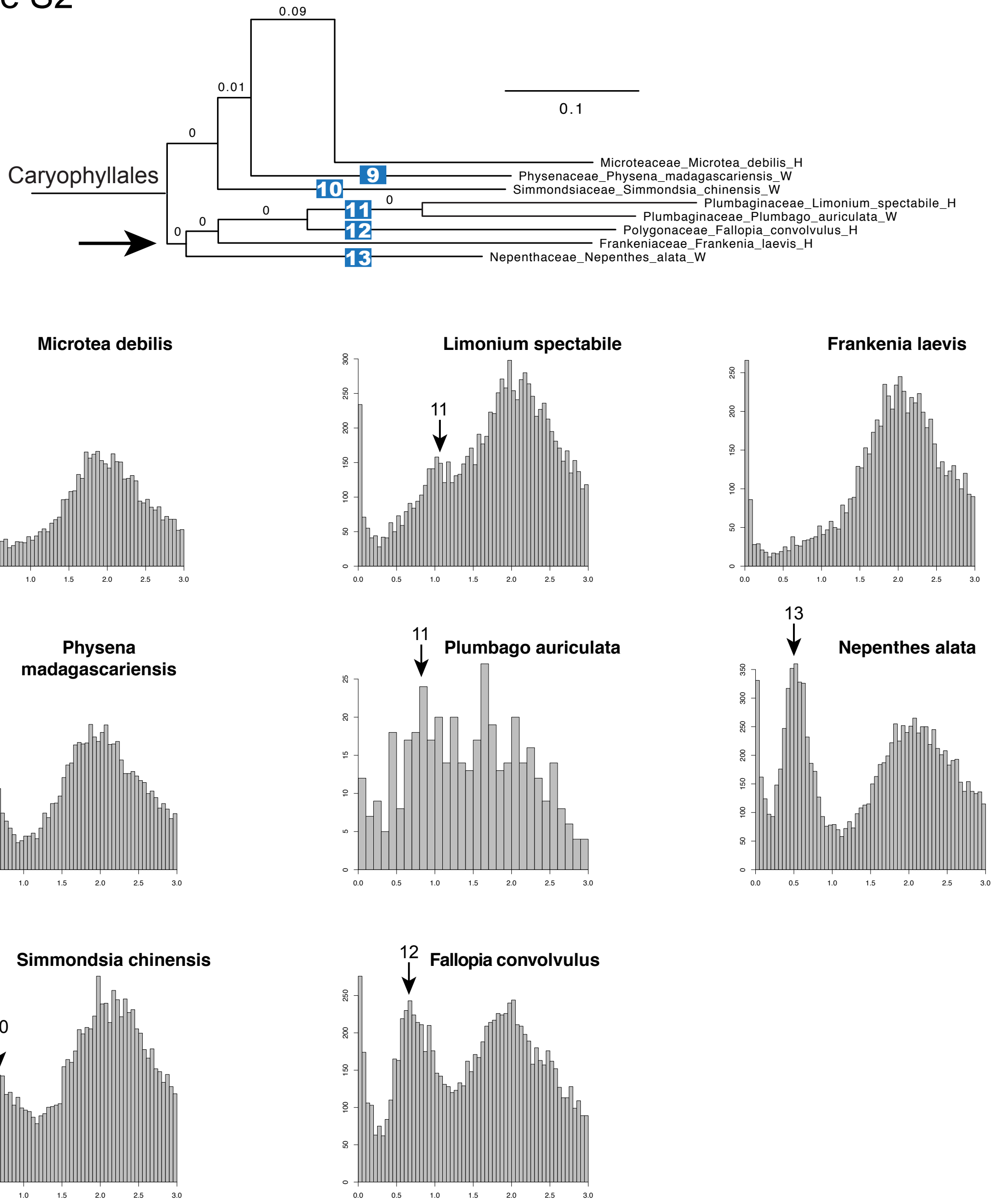
0.09
0.01
0
0.1
Caryophyllales
Microteaceae_Microtea_debilis_H
9
Physenaceae_Physena_madagascariensis_W
10
Simmondsiaceae_Simmondsia_chinensis_W
11
0
Plumbaginaceae_Limonium_spectabile_H
Plumbaginaceae_Plumbago_auriculata_W
0
0
0
12
Polygonaceae_Fallopia_convolvulus_H
Frankeniaceae_Frankenia_laevis_H
13
Nepenthaceae_Nepenthes_alata_W
Microtea debilis
Limonium spectabile
Frankenia laevis
Physena madagascariensis
Plumbago auriculata
Nepenthes alata
Simmondsia chinensis
Fallopia convolvulus

Fig. S3

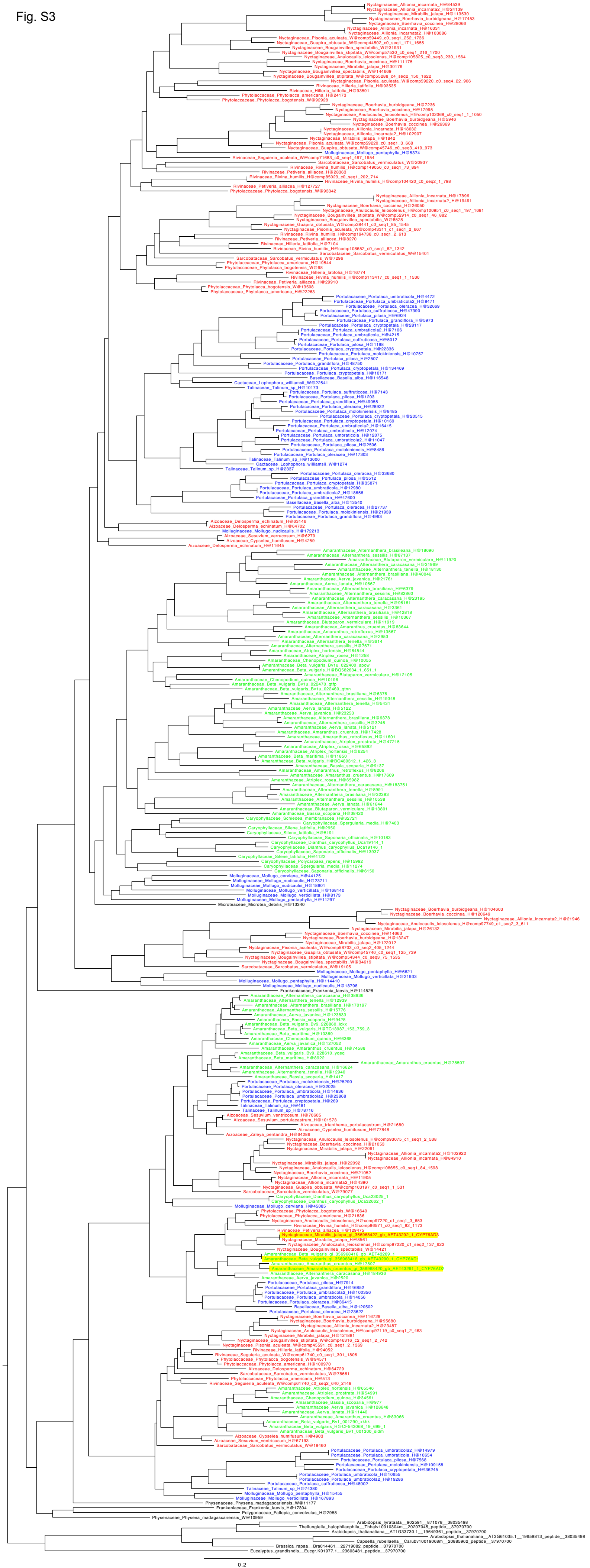

Supplement: Supplementary Data [file supp_msv081_supplementary.pdf]
